# Time-restricted feeding protects against septic liver injury by reshaping gut microbiota and metabolite 3-hydroxybutyrate

**DOI:** 10.1080/19490976.2025.2486515

**Published:** 2025-04-13

**Authors:** Jing-Juan Hu, Fan Deng, Qi-Shun Sun, Qing-Ming Xiong, Yue Min, Si-Yuan Feng, Ze-Bin Lin, Peng-Han Chen, Zhen Hu, Ling Wu, Xiao-Feng Chen, Sun Xie, Wei-Feng Liu, Cai Li, Ke-Xuan Liu

**Affiliations:** a Department of Anesthesiology, Nanfang Hospital, Southern Medical University, Guangzhou, Guangdong, China; b Guangdong Provincial Key Laboratory of Precision Anesthesia and Perioperative Organ Protection, Guangzhou, Guangdong, China; c Department of Anesthesiology, The First People’s Hospital of Foshan, Foshan, China

**Keywords:** Time-restricted feeding, *Lactobacillus murinus*, 3-hydroxybutyrate, PI3K/AKT/mTOR/LPIN1 pathway, ferroptosis, septic liver injury

## Abstract

Liver injury is an independent risk factor for multiple organ dysfunction and high mortality in patients with sepsis. However, the pathological mechanisms and therapeutic strategies for sepsis-associated liver injury have not been fully elucidated. Time-restricted feeding (TRF) is a promising dietary regime, but its role in septic liver injury remains unknown. Using 16S rRNA gene sequencing, Q200 targeted metabolomics, transcriptomics, germ-free mice, Hmgcs2/Lpin1 gene knockout mice, and Aml12 cells experiments, we revealed that TRF can mitigate septic liver injury by modulating the gut microbiota, particularly by increasing *Lactobacillus murinus* (*L. murinus*) abundance, which was significantly reduced in septic mice. Further study revealed that live *L. murinus* could markedly elevate serum levels of metabolite 3-hydroxybutyrate (3-HB) and alleviate sepsis-related injury, while the knockout of the key enzyme for 3-HB synthesis (3-hydroxy-3-methylglutaryl-CoA synthase 2, Hmgcs2) in the liver negated this protective effect. Additionally, serum 3-HB levels were significantly positively correlated with *L. murinus* abundance and negatively correlated with liver injury indicators in septic patients, demonstrating a strong predictive value for septic liver injury (AUC = 0.8429). Mechanistically, 3-HB significantly inhibited hepatocyte ferroptosis by activating the PI3K/AKT/mTOR/LPIN1 pathway, reducing ACSL4, MDA, LPO, and Fe^2+^ levels. This study demonstrates that TRF reduces septic liver injury by modulating gut microbiota to increase *L. murinus*, which elevates 3-HB to activate PI3K/AKT/mTOR/LPIN1 and inhibit hepatocyte ferroptosis. Overall, this study elucidates the protective mechanism of TRF against septic liver injury and identifies 3-HB as a potential therapeutic target and predictive biomarker, thereby providing new insights into the clinical management and diagnosis of septic liver injury.

## GRAPHICAL ABSTRACT

**Figure:**
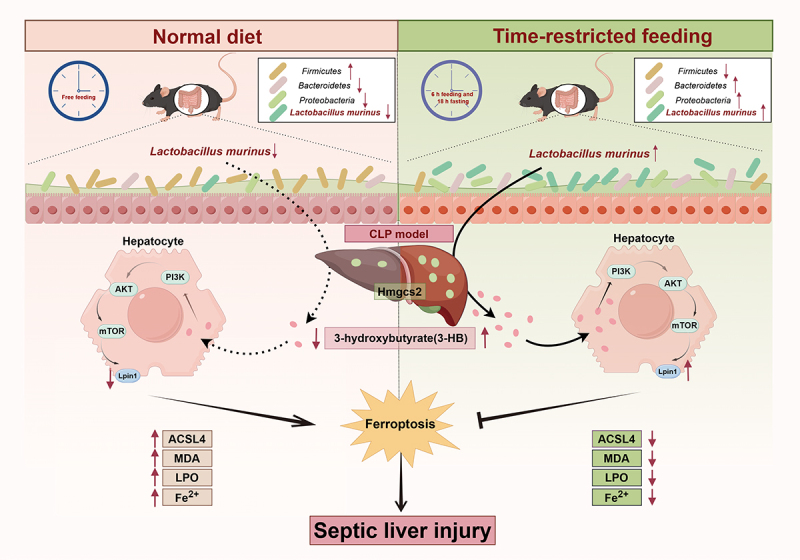

## Introduction

Sepsis, a severe condition caused by an uncontrolled response to infection, leads to multi-organ dysfunction^1–3^ and is particularly damaging to the liver, affecting patient outcomes and mortality rates in intensive care units.^4^ Current treatments for sepsis primarily involve antibiotics and organ support therapy, yet no effective drugs specifically target septic liver injury. Therefore, it is imperative to explore new therapeutic and preventive strategies to alleviate septic liver injury and enhance the quality of life for survivors.

Dietary patterns significantly influence lifespan and overall health.^5^ Time-restricted feeding (TRF) is an emerging dietary regimen that has garnered attention for its ability to counteract diet-induced obesity, improve insulin sensitivity, and reduce chronic inflammation.^6–8^ However, the mechanisms and effects of TRF on septic liver injury have not been reported. The gut microbiota, residing throughout various parts of the human gastrointestinal tract, forms a complex, dynamic, and diverse microbial community that is crucial for maintaining host homeostasis and supporting various physiological functions.^9^ Dysbiosis of the gut microbiota is closely linked to multiple diseases, including sepsis. In sepsis patients, liver dysfunction and metabolic disorders further exacerbate gut microbiota dysbiosis and damage the intestinal barrier, leading to multiple organ dysfunction syndrome.^10^ Although the influence of intestinal commensal bacteria and their metabolites on multiorgan damage in sepsis has been investigated, their exact role in sepsis pathogenesis remains unclear.^11,12^ Further studies are needed to understand the potential of intestinal microbiota and its metabolites as predictors, diagnostic biomarkers, and preventive treatments for sepsis and the associated organ damage. Although TRF influences disease development by altering the gut microbiota,^13,14^ its potential to mitigate sepsis-induced liver injury via the gut-liver axis remains underexplored.

In this study, TRF alleviates septic liver injury by reshaping the gut microbiota, significantly increasing *Lactobacillus murinus* (*L. murinu*s) abundance. Furthermore, we found that *L. murinu*s abundance was significantly reduced in septic mice. *L. murinus*, a gram-positive bacterium from the *Lactobacillaceae* family, performed beneficial functions such as inhibiting salt-sensitive hypertension, promoting colonic epithelial cell proliferation, alleviating colitis, and enhancing intestinal barrier function.^15–18^ These effects collectively contribute to reducing systemic inflammation, decreasing bacterial translocation, and maintaining intestinal homeostasis, all of which are critical for preventing and managing sepsis. Additionally, *L. murinus* alleviates intestinal ischemia-reperfusion injury by promoting the production of IL-10 in macrophages through TLR2 signaling and complications associated with delayed-onset infections in neonates.^19,20^ These findings suggest that *L. murinus* may play a significant role in treating sepsis, particularly in reducing secondary organ damage and mitigating inflammatory responses. However, despite these promising indications, the specific actions and mechanisms of *L. murinus* in sepsis remain unclear.

Metabolomics analyses revealed that *L. murinus* treatment significantly increased 3-hydroxybutyrate (3-HB) levels in the cecum of mice. 3-HB, synthesized in the liver via fatty acids β-oxidation, serves as an alternative energy source during energy deficiency. The therapeutic potential of 3-HB has attracted interest, particularly in conditions, such as neurodegenerative diseases, alcoholic fatty liver, ischemia-reperfusion injury, and atherosclerosis,^21–23^ through mechanisms involving reduced inflammation, apoptosis, oxidative stress, and fibrosis. However, its specific role in septic liver injury remains unknown.

The PI3K/AKT/mTOR pathway plays a crucial role in inhibiting apoptosis, inflammatory responses, and reducing sepsis mortality.^24^ However, its precise involvement in sepsis remains controversial. Some studies suggest that blocking this pathway alleviates sepsis-related damage,^25,26^ whereas others indicate that activating this pathway reduces liver injury.^27^ Interestingly, the Chinese medicine Xuebijing has shown a dual effect of activating and inhibiting this pathway at different stages of sepsis.^28^ In the current study, pre-treatment with 3-HB enhanced the activation of the PI3K/AKT/mTOR pathway in cecal ligation and puncture (CLP) mice, although the specific mechanisms require further investigation. Lpin1, a downstream target of the PI3K/AKT/mTOR pathway,^29^ is implicated in lipid synthesis, the sepsis immune response, and ferroptosis.^30–32^ Ferroptosis, a type of non-apoptotic cell death that is dependent on intracellular iron accumulation, contributes to septic liver injury.^33^ 3-HB affected ferroptosis and the PI3K/AKT/mTOR pathway in various disease models.^34,35^ However, the mechanisms through which 3-HB inhibits hepatocyte ferroptosis and mitigates liver injury during sepsis via the PI3K/AKT/mTOR pathway remain unknown.

In this study, we aimed to explore the mechanisms and effects of TRF on septic liver injury from the perspective of the gut microbiota. Our findings reveal that live *L. murinus* could markedly elevate serum levels of metabolite 3-HB and alleviate septic liver injury, while the knockout of the key enzyme for 3-HB synthesis (3-hydroxy-3-methylglutaryl-CoA synthase 2, Hmgcs2) in the liver negated this protective effect. 3-HB activates the PI3K/AKT/mTOR/LPIN1 signaling pathway, thereby inhibiting hepatocyte ferroptosis and ultimately reducing septic liver injury. This study reveals the protective mechanism of TRF against septic liver injury and identifies 3-HB as a potential therapeutic target and predictive biomarker, offering new approaches for clinical treatment and diagnosis of septic liver injury.

## Materials and methods

### Human study populations

Patients were recruited from the ICU of the First Peoples Hospital of Foshan, Guangdong, China, between June 2022 and April 2023. The SOFA score was assessed on the first day of ICU admission, and clinical data throughout hospitalization was recorded. All research was conducted in accordance with both the Declarations of Helsinki and Istanbul. This study received approval from the Ethics Committee of the First Peoples Hospital of Foshan, and written informed consent was obtained from the patients (Ethics approval number: FSYYYEC-2021-106).

Blood analysis was performed on patients with sepsis (defined as infection + SOFA score ≥ 2) upon admission. Patients with sepsis who fulfilled at least one or more of the following three conditions (ALT ≥ 80 U/L, total bilirubin ≥34 µmol/l or AST ≥ 100 U/L) were defined as sepsis liver injury (SLI) group; while those who did not meet the diagnostic criteria for sepsis liver injury were defined as the sepsis non-liver injury (SNLI) group. Exclusion criteria included hospital stay <1 d, chronic liver disease, pharmacological liver injury, combined metabolic or autoimmune disease, and obstructive jaundice. Based on the inclusion and exclusion criteria, a total of 57 patients with sepsis were included in this study, including 17 patients with SLI and 40 patients with SNLI. Serum and fecal samples were collected for laboratory tests on the day of ICU admission.

### Animal

Specific pathogen-free C57BL/6J male mice (6–8 weeks old, 20–25 g) were purchased and raised at the animal center of Nanfang Hospital, Southern Medical University. Germ-free (GF) mice were purchased from Shenzhen Jingtuo Biotechnology Co., Ltd (Shenzhen, China). Lpin1^−/−^ mice were purchased from Cyagen Biosciences Company (Suzhou, China). All experimental animals were housed and handled in compliance with the guidelines established by the National Institutes of Health, and all procedures were approved by the Animal Care and Use Committee of Nanfang Hospital, Southern Medical University (Guangzhou, China) (approval number IACUC-LAC-20220616-006). Mice were kept under controlled temperature, humidity, and 12-h light/dark cycles with *ad libitum* access to food and water.

### Mouse model of sepsis

Cecal ligation and puncture (CLP)-induced severe sepsis model was established according to our previous study.^12^ Briefly, mice were anesthetized with isoflurane and secured. The abdomen was shaved, disinfected, and incised along the midline. The distal 3/4 of the cecum was ligated, punctured, and its contents extruded before suturing the muscle layer and skin. Postoperative fluid resuscitation was performed. The sham group only underwent laparotomy without ligation and puncture.

Another sepsis model was induced by intraperitoneal injection of LPS (Sigma-Aldrich, USA) at a dose of 10 mg/kg. Mice were sacrificed 12 h after CLP operation or LPS injection for detection organ damage.

### Time-restricted feeding experiment

To investigate the impact of time-restricted feeding (TRF) on sepsis injury, this study implemented a specific TRF pattern: mice were allowed fed ad libitum for 6 h/restricted diet for 18 h daily from 9:00 to 15:00, with free access to water for 2 weeks. C57BL/6J male mice aged 6–8-weeks were randomly divided into four groups: (1) normal diet (ND) + sham group (ND + Sham), (2) TRF + sham group (TRF+Sham), (3) ND + CLP group and (4) TRF + CLP group. Bedding was changed daily and weight changes were recorded. The mice were anaesthetized 12 h after surgery and heart blood, cecum, liver, lung and kidney tissues were collected for further study (6–8 mice per group). In addition, survival rates of mice in the above groups were monitored for 5 days following the CLP operation (*n* = 20). These mice were purchased from the same batch of the same manufacturer.

## Antibiotic treatment

Pseudo germ-free mouse model experiment established by antibiotic (ABX) treatment. C57BL/6J mice aged 6–8-weeks were randomly divided into four groups: (1) ND+CLP group, (2) TRF+CLP group, (3) ND+ABX+CLP group: quadruple antibiotics (vancomycin 100 mg/kg, neomycin 200 mg/kg, metronidazole 200 mg/kg and ampicillin 200 mg/kg) were given intragastrically once daily in the first week and once every other day in the second week in a volume of 200 μl to deplete the gut microbiota) and (4) TRF+ABX+CLP group: Mice were given TRF and ABX for 2 weeks to establish the CLP model. Bedding was changed daily and weight changes were recorded (6–8 mice per group). Survival experiments with mice in the above groups were monitored for 5 days after CLP operation (*n* = 20).

## FMT experiment

6–8-week-old male C57BL/6J mice were given ABX intragastrically once daily for 1 week (receptor mice). Preoperatively collected feces from ND and TRF mice (donor mice) were resuspended in PBS at a concentration of 0.125 g/ml. A volume of 0.1 ml of this solution was delivered to mice in the respective groups via gavage for 1 week (6–8 mice per group). During this process, all mice were fed a TRF diet and had ad libitum access to water. Mice were performed CLP surgery after 1 week of transplantation, and then blood, cecum, kidney, lung, and liver samples were harvested in a sterile manner for further examination. These mice were purchased from the same batch of the same manufacturer.

## Bacterial strains and growth conditions

The recovery and activation conditions of *L. murinus* were referred to our previous study.^19^ In brief, frozen stocks of *L. murinus* (ATCC35020) were prepared and stored at −80°C for further experiments. A 50 μl *L. murinus* suspension was added to 5 ml MRS medium and then incubated at 37°C under anaerobic conditions for 18 h. Subsequently, 200 µL live *L. murinus* (6.8 × 10^9^ CFU/mL) were administered to mice via gavage. In addition, the Killed *L. murinus* was treated by inactivating the live *L. murinus* suspension at 70°C for 30 min.

## GF mice experiment

Eighteen male C57BL/6J GF mice, aged 6–8 weeks, were randomly and equally assigned to three groups: (1) ND+CLP group, (2) TRF+CLP group and (3) *L. murinus*+TRF+CLP group: mice were gavaged with 200 µL live *L. murinus* (6.8 × 10^8^ CFU/mL) for 1 week. During this process, bedding was changed daily and weight changes were recorded. Twelve hours after CLP surgery, mice serum, cecal contents and liver samples were gathered for further examination.

## 16S rRNA gene sequencing

Microbial DNA was extracted using the HiPure Stool DNA Kits (Magen, Guangzhou, China) according to the manufacturer’s protocols. The hypervariable region V3–V4 of the bacterial 16S rRNA gene was amplified with primer by an ABI StepOnePlus Real-Time PCR System (Life Technologies, USA). All samples were paired-end sequenced on the Illumina (Hiseq PE250). Raw reads were then filtered using FASTP (version 0.18.0) and paired reads were overlapped as raw tags using FLASH (version 1.2.11). Noisy sequences of raw tags were filtered based on specific filtering condition**s** to obtain the high-quality clean tags. These clean tags were then clustered into operational taxonomic units (OTUs) with a similarity of ≥97% using the UPARSE^36^ pipeline (version 9.2.64). Based on OTU sequences and abundance, community composition analysis, indicator species analysis, alpha and beta diversity analysis and PICRUSt2 functional prediction analysis were performed.

## Quantitative real-time PCR analysis

Total RNA from cells or Mouse liver tissue RNA was extracted by TRIZOL reagent (#15596026, Invitrogen). A reverse transcript enzyme (TOYOBO, Tokyo, Japan) was applied to prepare cDNA according to the manufacturer’s protocol. The real-time PCR reaction was performed using the ABI Q6 real-Time PCR System with SYBR Green detection protocol (TOYOBO, Tokyo, Japan). The target gene primers are listed in Table S1. The relative mRNA expression level was calculated using the 2^−^^^CT analysis method. To assess the total *L. murinus* in cecum and stool samples, quantitative real-time PCR was performed using primers: *L. murinus*,^19^ LactoM-F (5′-TCGAACGAAACTTCTTTATCACC-3′), and LactoM-R (5′-CGTTCGCCACTCAACTCTTT-3′).

## Biochemical analysis

Endotoxin levels in mouse serum were measured using a commercial ELISA kit (CUSABIO, Wuhan, China) according to the manufacturer’s instructions. Serum alanine aminotransferase (ALT), aspartate aminotransferase (AST), creatinine and Blood Urea Nitrogen (BUN) levels in mice were determined manually with a commercial kit (KeyGene, Nanjing, China) according to the manufacturer’s instructions. The 3-HB level in mouse serum was measured using a commercial kit (GenScript, Nanjing, China). Additionally, 3-HB levels in patient serum were measured using a commercial kit (Elabscience® Biotechnology Co., Ltd).

## Protein extraction and western blot analysis

Cell or Mouse liver tissue proteins were extracted using a RIPA lysis buffer (Solarbio, Beijing, China). Then, the samples were resolved by 10% SDS-PAGE and transferred to polyvinylidene fluoride membranes (PVDF, Merck Millipore, Billerica, MA, USA). The membrane was blocked for 20 min at room temperature using Quick block buffer (Epizyme, Shanghai, China), after which primary antibodies were incubated with membranes overnight at 4°C, including PI3K (#67071–1-Ig, 1:5000, Mouse, Proteintech), AKT (#4691, 1:1000, rabbit, Cell Signaling Technology), p-AKT (#9271, 1:1000, rabbit, Cell Signaling Technology), mTOR (#2972,1:1000, rabbit, Cell Signaling Technology), p-mTOR (#2974, 1:1000, rabbit, Cell Signaling Technology), LPIN1 (#5195, 1:1000, rabbit, Cell Signaling Technology), GPX4 (#52455, 1:1000, rabbit, Cell Signaling Technology), ACSL4 (#DF12141, rabbit, 1:1000, Affbiotech) and GAPDH (10494–1-AP, 1:5000, rabbit, Proteintech). Then the membrane was incubated for 1 h at room temperature with the respective secondary antibodies Anti-rabbit IgG (#7074, 1:1000, Cell Signaling Technology). Bands were detected by the enhanced chemiluminescent liquid (ECL, #P0018AM, Beyotime Biotechnology, China) and images were captured using the ChemiDoc MP imaging system (BIORAD). Blotting analysis was performed with ImageJ (National Institutes of Health, Java 1.8.0).

## Histological staining

Liver, lung, and kidney tissues were collected and fixed in 4% paraformaldehyde for 48 h. Then, the samples were dehydrated, embedded in paraffin, sectioned at 4 μm thickness and stained with hematoxylin-eosin (HE) according to the experimental protocol. Histological damage was scored according to previously describe scoring system for liver, lung and kidney tissue.^37–39^ Eight randomly selected images from each sample (*n* = 6) were captured at × 200 using an Olympus fluorescence microscope (Olympus, Japan).

## Immunofluorescence

Paraffin sections of mouse liver tissue were prepared as described above. The samples underwent antigen repaired (EDTA, pH 9.0) and were subsequently blocked with 10% donkey serum for 20 min at room temperature. They were then incubated overnight at 4°C with anti-HMGCS2 antibody (1:100) (Abcam, Cambridge, MA, USA) and anti-ACSL4 antibody (1:100) (Proteintech). Then the liver tissue was washed and stained with DAPI for 5 min. Fluorescence Images were captured by fluorescence microscopy (Olympus, Japan). Quantification of the fluorescence intensity of HMGCS2 and ACSL4 staining in the liver tissue were carried out using ImageJ (National Institutes of Health, Java 1.8.0) in 6 randomly selected fields at 200× magnification for each sample.

## Q200 macrometabolomic assays and data analysis

Cecum samples were prepared according to the Metabo-Profile Biotechnology (Shanghai) Co. Ltd‘s instructions. An ultra-performance liquid chromatography coupled to tandem mass spectrometry (UPLC-MS/MS) system (ACQUITY UPLC-Xevo TQ-S, Waters Corp., Milford, MA, USA) was used to quantitate the metabolite. The chromatographic columns used in this experiment were ACQUITY UPLC BEH C18 1.7 μM VanGuard pre-column (2.1 × 5 mm) and ACQUITY UPLC BEH C18 1.7 μM analytical column (2.1 × 100 mm). Raw data files from UPLC-MS/MS were processed using MassLynx software (v4.1, Waters, Milford, MA, USA) for peak integration, calibration, and quantitation of each metabolite. Orthogonal partial least square discriminant analysis (OPLS-DA) was used for multi-class classification and identification of differently altered metabolites. Metabolic pathway analysis was performed using the MetaboAnalyst website (http://www.metaboanalyst.ca/) to uncover metabolic pathways associated with differential metabolites. The self-developed platform iMAP (v1.0, Metabo-Profile, Shanghai, China) was used for statistical analyses, including PCA, OPLS-DA, univariate analysis and pathway analysis.

## Quantitative analysis of 3-HB

LC-MS/MS was used to quantify the levels of the metabolite 3-HB in the bacterial supernatant after 12 h anaerobic incubation of *L. murinus* and in the supernatant of the MRS medium, as well as in the feces of WT, GF mice and GF+*L. murinus* treated mice. Based on previous studies, samples were pretreated by protein precipitation. Solutions of 3-HB standards at different concentrations (1 ng/ml, 5 ng/ml, 20 ng/ml, 100 ng/ml, 500 ng/ml and 1000 ng/ml) were prepared for analysis. Chromatographic separation was performed on a Thermo Scientific Prelude SPLC system, which integrates an on-line sample cleanup and liquid-phase system. Analysis was conducted with a TSQ Vantage triple quadrupole mass spectrometer and Prelude SPLC system from Thermo Fisher Scientific. The chromatographic column utilized was a Waters BEH C18 2.1 × 100 mm, 1.7 μm; with mobile phases A: 10 mm ammonium formate adjusted to pH 3.0 and B: methanol. The column temperature was maintained at 50°C, and an injection volume of 5 μl was used. Data acquisition and processing were executed using TraceFinderTM software (version 3.3 sp1, Thermo Fisher Scientific, USA).

## 3-HB treatment experiment

C57BL/6J male mice were administered at different concentrations^40,41^ (50 mg/kg, 100 mg/kg and 200 mg/kg) of 3-HB sodium salt 10 min prior to LPS and CLP surgery. In addition, mice were treated with 45 mg/kg PI3K/AKT/MTOR signaling pathway inhibitor NVP-BEZ235 and 100 mg/kg 3-HB sodium salt respectively, 1 h and 10 min before the surgery, in order to further verify and explore the activation of PI3K/AKT/MTOR signaling pathway by 3-HB to reduce septic liver injury. To further verify and explore the role of the PI3K/AKT/mTOR pathway in inhibiting ferroptosis to reduce septic liver injury, mice were treated with 45 mg/kg NVP-BEZ235 and 5 mg/kg ferrostatin-1 (Fer) respectively, 1 h and 30 min before the surgery.

## RNA-seq sequencing

Total RNA from Mouse liver tissue RNA was extracted by TRIzol reagent (#15596026, Invitrogen). The cDNA fragments were purified with QiaQuick PCR extraction kit (Qiagen, Venlo, Netherlands). The ligation products were size selected by agarose gel electrophoresis, PCR amplified, and sequenced using Illumina novaseq 6000 by Gene Denovo Biotechnology Co. (Guangzhou, China). High quality reads were filtered using fastp prior to conducting HISAT2 alignment for transcript reconstruction with Stringtie, and gene expression calculation with RSEM. Subsequently, PCA and correlation analyses were performed to investigate sample relationships. Gene differential expression analysis was carried out using edgeR software, involving read count normalization, hypothesis testing probability calculation, and FDR value correction for multiple hypothesis testing. Genes with FDR < 0.05 and |log2FC|>1 was identified as significantly different. Following this, Gene Ontology and KEGG enrichment analyses were conducted on the differentially expressed genes, with pathways having a Qvalue ≤ 0.05 considered significantly enriched. These pathways provide insights into the biochemical metabolism and signal transduction processes involving the differentially expressed genes.

## Mouse Hmgcs2 knockout Adeno-Associated Virus (AAV) packaging project

The mouse Hmgcs2 knockout AAV packaging project was carried out by Cyagen Biosciences Company (Suzhou, China). The mouse Hmgcs2 gene (NCBI Reference Sequence: NM_008256.4, Ensembl: ENSMUSG00000027875) is located on mouse chromosome 3. A total of 10 exons are identified, with the ATG start codon in exon 1 and the TAA stop codon in exon 9 (Transcript Hmgcs2–201-ENSMUST00000090746.3). Exon 4 will be selected as target site. A Single-gRNA AAV knockout vector targeting mouse hmgcs2 was created using a Scaffold sequence that aligns with SaCas9. The SgHmgcs2 group plasmid was constructed as pAAV-U6 > {mHmgcs2-SagRNA}-TBG>SaCas9, while the Control group plasmid was pAAV-TBG-EGFP. Both the Single-gRNA vector and Control vector were packaged with AAV8 for titer detection to ensure that the virus quality meets the required standards for subsequent experiments (titer ≥1E + 13 vg/ml).

Wild-type C57BL/6J mice were injected with 100 µL (1 × 10^^12^ vg/ml) of Hmgcs2 gene knockout virus (AAV8-sgHmgcs2) and control virus (AAV8-Ctrl) via tail vein. After 28 days, a CLP model was established. At day 21 post-virus injection, mice in the CLP+*L. murinus* group were orally administered live *L. murinus* (6.8 × 10^8^ CFU/mL) for 1 week. Additionally, C57BL/6J mice were intraperitoneally injected with 1 mg/kg Hymeglusin, an Hmgcs2 inhibitor, to investigate the impact of *L. murinus* on 3-HB production in septic liver injury mediated by Hmgcs2.

## Mouse Lpin1 knockout AAV packaging project

The mouse Lpin1 knockout AAV packaging project was conducted by Cyagen Biosciences Company (Suzhou, China). The Mouse Lpin1 gene is located on Mouse chromosome 12 (NCBI Reference Sequence: NM_172950.3, Ensembl: ENSMUSG00000020593). The SgLpin1 group plasmid was constructed as pAAV-U6>{mLpin1-SagRNA}-TBG>SaCas9, while the Control group plasmid was pAAV-TBG-EGFP. The plasmid was transfected with pVSV-G and pSPAX2 into the HEK293T cell line. The lentivirus-containing supernatant was collected 48–72 h post-transfection, followed by ultracentrifugation and then infection of the Mouse cell line. After 48–72 h of infection, Puro was added for selection of the cells under stress. One week after screening, cells were collected, genomes were extracted, and PCR was performed to screen for gRNA with the best knockout efficiency. The SgRNA vector and Control vector were packaged with AAV8 for titer detection to ensure that the virus quality met the requirements of subsequent experiments (titer ≥1E + 13vg/ml).

Wild-type C57BL/6J mice were injected with 100 μL (1 × 10^^12^ vg/ml) Lpin1 gene knockout AAV virus (AAV8-sgLpin1) and control AAV virus (AAV8-Ctrl) via the tail vein for a duration of 4 weeks, resulting in peak interference effect. Subsequently, the mice were divided into two groups: (1) CLP group and (2) CLP + 3-HB group: C57BL/6J male mice received 100 mg/kg 3-HB sodium salt 10-min prior to CLP surgery.

## Mouse Lpin1 interference (LV) packaging project

A shRNA LV vector targeting mouse Lpin1 (ID: 14245, NCBI RefSeq transcripts: NM_001130412.1) was conducted by Cyagen Biosciences Company (Suzhou, China). The control vector used was LV-U6>Scramble-shRNA-PGK>Puro, with the shRNA Sequence: Scramble shRNA. The shRNA Vector 1 used was: LV-U6>mLpin1[shRNA#1]-PGK>Puro, with the shRNA Sequence: shRNA #1 GCCGTGTCATATCAGCAATTTCTCGAGAAATTGCTGATATGACACGGC. shRNA Vector 2 : LV-U6>mLpin1[shRNA#2]-PGK>Puro, shRNA Sequence: shRNA #2 CTTGACGCGACCGAGCATTAACTCGAGTTAATGCTCGGTCGCGTCAAG; shRNA Vector 3 : LV-U6>mLpin1[shRNA#3]-PGK>Puro， shRNA Sequence: shRNA #3 AGTAAGGCCCAGACGGAAATGCTCGAGCATTTCCGTCTGGGCCTTACT. Following the extraction of high-purity endotoxin-free plasmid, the vector plasmid containing the target gene or shRNA, along with the two helper plasmids psPAX2 and pMD2G, were co-transfected into 293T cells using LipofiterTM transfection reagent. The virus supernatant was collected within 48–72 h post-transfection, concentrated, and purified via ultracentrifugation to yield a high-titer lentivirus preservation solution. The final measured lentivirus titers were as follows: shRNA#1: 1.19 × 10^9^ TU/ml, shRNA#2: 4.94 × 10^8^ TU/ml, shRNA#3: 3.41 × 10^8^ TU/ml, control virus: 9.38 × 10^8^ TU/ml.

## Cell line and culture conditions

The study utilized liver parenchymal cells (AML12 cells) derived from human TGF-α transgenic mice (CD1 and MT42 strains). The cells were cultured in a specialized medium under controlled conditions of 5% CO_2_, 37°C, and saturated humidity. The culture medium was refreshed after the initial 36 h and then every 24 h to maintain cell viability for further experiments. Prior to LPS stimulation, the cells were pre-treated with 1 μM of the signaling pathway inhibitor NVP-BEZ235 and 10 mm of 3-HB sodium salt,^42^ administered 1 h and 4 h before stimulation, respectively. Prior to LPS stimulation, the cells were pre-treated with 1 μM of NVP-BEZ235 and 500 nM of Fer, administered 1 h and 30 min before stimulation, respectively. After 24 h 100 ng/ml LPS stimulation, both AML12 cells and the supernatant were collected for analysis.

## Cell transfection

Cell transfection involved seeding 2 × 10^6^ AML12 cells in a 12-well cell culture plate, incubating them in a carbon dioxide incubator at 37°C and 5% CO2 for 15–20 h. Subsequently, the virus liquid (shRNA1, shRNA2, shRNA3) was melted in an ice bath, added to an appropriate amount of culture medium based on MOI values of 1, 10, and 100, and mixed thoroughly. To enhance infection efficiency, 5 μg/ml Polybrene was added to the infected cells before placing them back in the incubator overnight. After removing the virus-containing medium, fresh complete medium was added, and the cells were cultured further in the same incubator conditions. Typically, genes carried by lentiviral vectors start expressing after approximately 24 h. Following transfection of Lpin1 gene shRNA2 lentivirus and control shRNA lentivirus into AML12 cells using the aforementioned protocol, the cells were divided into two groups: (1) LPS group and (2) LPS + 3-HB group (AML12 cells pre-treated with 10 mm 3-HB sodium salt for 4 h).

## Hmgcs2 overexpression AAV packaging project

The mouse Hmgcs2 overexpression AAV packaging project was carried out by Cyagen Biosciences Company (Suzhou, China). The AAV overexpression vector was designed to express the mouse Hmgcs2 gene (NM_008256.4) under the control of the liver-specific TBG promoter. The Hmgcs2 overexpression plasmid was constructed as AAV-TBG>Kozak-Mouse Hmgcs2 CDS[NM_008256.4]/3×FLAG/P2A/EGFP. The control group plasmid was constructed as pAAV-TBG-EGFP. The AAV8 was packaged using the overexpression vector, and the viral titer was determined to ensure the quality met the requirements for subsequent experiments (titer ≥1E + 13 vg/ml 1 × 10^13^ vg/mL).

AML12 cells were seeded in 6-well plates (2 × 10^5^ cells/well) and cultured in DMEM/F12 medium with 10% FBS and 1% penicillin/streptomycin at 37°C under 5% CO₂. Cells were transfected with AAV8-Hmgcs2 or AAV8-Ctrl (MOI = 1 × 10^4^ vg/cell) in serum-free medium for 6 h, followed by replacement with complete medium and further culture for 48 h. Transfected cells were divided into: (1) NC group (2) LPS group (100 ng/mL LPS, 24 h). After treatment, cell supernatants and pellets were collected for analysis.

## Cell viability assay

AML12 cell viability was assessed using the CCK8 detection kit (KeyGene, Nanjing, China) and lactate dehydrogenase (LDH) detection kit (KeyGene, Nanjing, China) according to the provided instructions.

## Ferroptosis-related indicators assay

The levels of MDA, LPO, and Fe^2+^ in liver tissue and AML12 cells were assessed using the malondialdehyde (MDA) content detection kit, lipid peroxide (LPO) content detection kit, and iron content detection kit (Solarbio, Beijing, China). The glutathione content in liver tissue and AML12 cells were determined using the total glutathione/oxidized glutathione (T-GSH/GSSG) test kit (KeyGene, Nanjing, China).

## SPR experiment

An SPR experiment was conducted to determine 3-HB binding affinity to recombinant human (rh) protein of rhPIK3R3 (Sf9, His, GST) (HY-P701744, MedChemExpress). 2 μl of 0.75 mg/mL recombinant protein was immobilized on a protein chip and activated with 10 mm NiSO4. Different concentrations of 3-HB (40, 80, 100 and 400 μM) were diluted with Dulbecco’s PBS (DPBS) and added to the mobile phase at a rate of 30 μm/min. The binding signal was recorded by PlexArray HT specialized software (Plexera Bioscience, USA). Data were analyzed using Origin 9.0 (OriginLab, USA) and BIAevaluation 4.1.1.

## Statistical analysis

Statistics and mapping were performed by GraphPad Prism 9. Statistical tests included Welch’s t test, one-way ANOVA, or two-way ANOVA as specified in the figure legends, with Tukey’s test applied for multiple group comparisons. The log-rank statistics were used to evaluate the survival curves of septic mice. Spearman’s rank correlation test was used to analyze the correlation. Clinical measurement data are expressed as (x±s) and t-test was performed, while count data are expressed as (%) and χ^2^ test was performed for intergroup comparison. Results are expressed as mean ± standard error of mean (SEM) or the median and quartile. A *p* value of <0.05 is considered significant, and the statistical sample size (n) is listed in each figure legend.

## Results

### Time-restricted feeding attenuates sepsis damage and modifies gut microbiota composition and diversity

**Figure 1.**
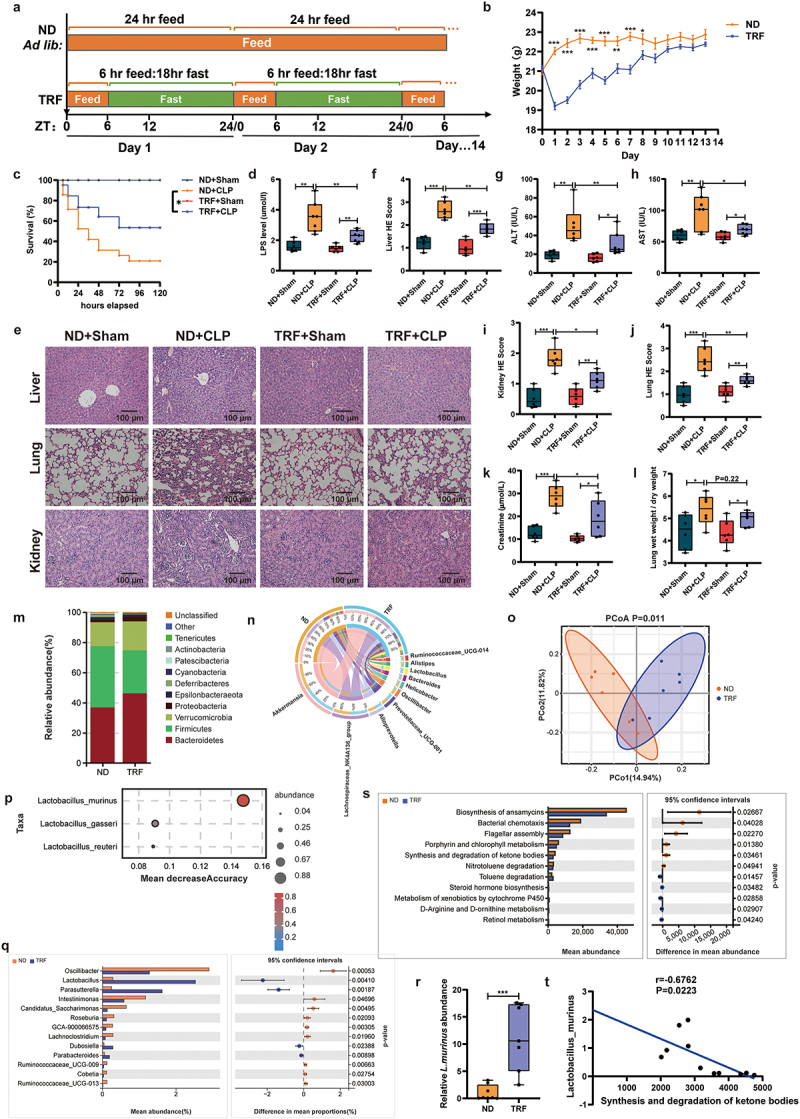
Time-restricted feeding attenuates sepsis damage and modifies gut microbiota composition and diversity. (a) Time-restricted feeding (TRF) pattern illustration. (b) Weight changes in ND and TRF mice within 14 days, *n* = 6–8. (c) 5-day survival of mice (*n* = 20). (d) Serum LPS levels, *n* = 6–8. (e) HE staining of liver, lung and kidney tissues of mice, scale: 100 μm, *n* = 6. (f) Liver histopathological damage scores, *n* = 6. (g, h) serum ALT and AST levels, *n* = 6–8. (i, j) Kidney and lung histopathological damage scores, *n* = 6. (k) Creatinine content in serum of mice, *n* = 6–8. (l) Mouse lung wet/dry weight ratio, *n* = 6. (m) Species distribution at the phylum level, *n* = 6. (n) Circos plot of species abundance at the genus level, *n* = 6. (o) PCoA analysis of microbial β diversity, *n* = 6. (p) Mean decrease accuracy in species random forest analysis at the species level, *n* = 6. (q) Statistical test analysis of indicator species at the genus level, *n* = 6. (r) Relative abundance of *L. murinus* in feces detected by qPCR, *n* = 6–8. (s) KEGG pathway analysis, *n* = 6. (t) Correlation between *L. murinus* abundance and synthesis and degradation of ketone bodies pathway (Spearman correlation analysis). The results are expressed as the mean ± SEM (B) and the median and quartile. * *p* < 0.05, ** *p* < 0.01, *** *p* < 0.001 by log-rank test (C), two-way ANOVA (Tukey’s test) (D, F-L) and welch's t test (S, R). ND: normal diet; TRF: time-restricted feeding; ALT: alanine Aminotransferase; AST: aspartate aminotransferase; *L. murinus*: *lactobacillus murinus*; PCoA: principal coordinate Analysis; qPCR: real-time quantitative PCR. SEM, standard error of mean.

C57BL/6J mice were subjected to a TRF regimen for 2 weeks (6 h feeding, 18 h fasting) before establishing the CLP sepsis model (Figure 1(a)). No significant difference was observed in the pre-modeling body weights of the mice (Figure 1(b)). TRF dramatically improved CLP mice survival, reduced serum LPS levels, and alleviated liver, lung, and kidney injuries, though the lung wet/dry weight ratio and serum BUN content remained unchanged (Figures 1(c-i) and and S1a). 16S rRNA gene sequencing of preoperative fecal samples revealed that TRF increased the abundance of *Bacteroidetes*, *Proteobacteria* and *Lactobacillus*, while decreasing *Firmicutes* abundance compared with the ND group (Figures 1(m,n) and S1b). Principal co-ordinates analysis (PCoA) results of β diversity indicated significant differences in the intestinal flora of ND and TRF mice (Figure 1(o)). No significant differences in α diversity were observed (Figures S1c-f). Notably, the *Lactobacillus* and *L. murinus* abundance in the TRF group increased significantly, and random forest analysis indicated that *L. murinus* was the strongest indicator between the two groups (Figures 1(p,q) and S1g). Furthermore, the relative abundance *L. murinus* in the feces of the two groups of mice was verified by qPCR (Figure 1(r)). PICRUSt2 prediction highlighted significant differences in the biosynthesis of ansamycin and synthesis and degradation of ketone bodies pathways, with the latter significantly correlated with *L. murinus* abundance (Figure 1(s,t)). These results indicate that TRF significantly altered gut microbiota composition and diversity.

### *TRF alleviates septic liver injury by reshaping the gut microbiota, especially* L. murinus

**Figure 2.**
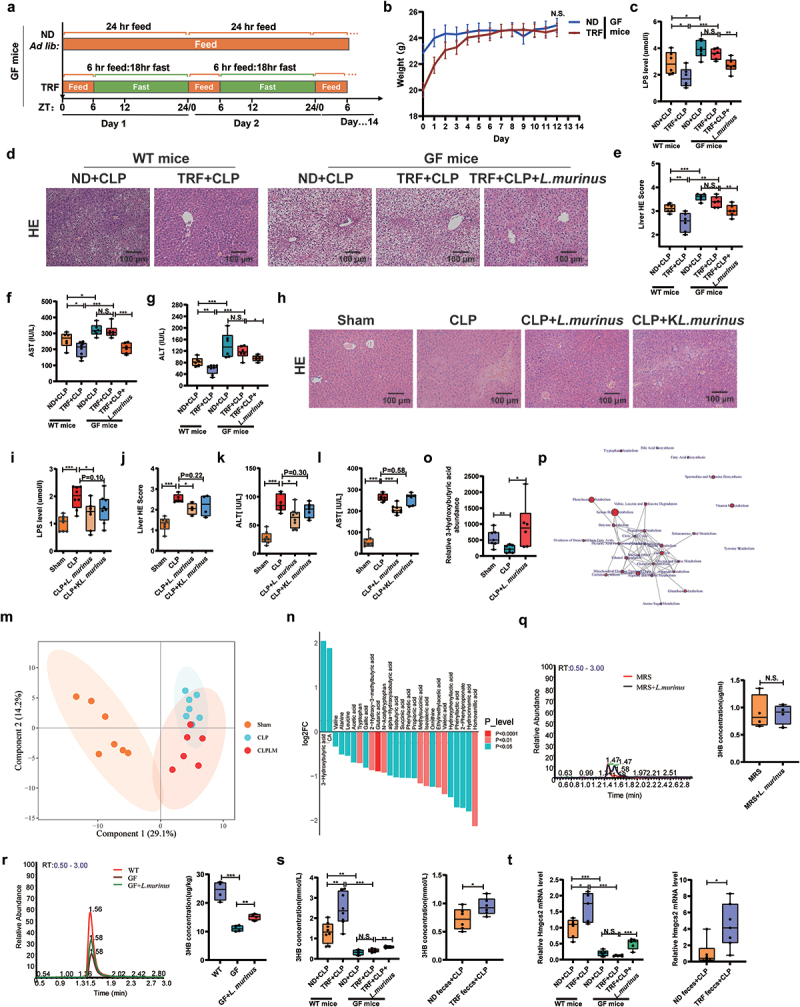
TRF alleviates septic liver injury by reshaping the gut microbiota, especially *L. murinus*. (a) Pattern plots of TRF and ND in GF mice. (b) Weight changes of GF mice in the TRF group and the ND group within 14 days, *n* = 6. (c) LPS content in serum of mice, *n* = 6. (d, e) HE staining and quantification analysis in liver, scale: 100 μm, *n* = 6. (f, g) Serum ALT and AST levels in mice, *n* = 6. (h, j) HE staining of liver tissues of mice in sham group, CLP group, CLP+*L. murinus* group and CLP+K*L. murinus* group and the quantification analysis, scale: 100 μm, *n* = 6. (i, k, l) Serum LPS, ALT and AST levels, *n* = 6–8. (m) Principal component analysis (PCA) of sample metabolites, *n* = 6–8. (n) Differential metabolites in cecal contents, *n* = 6–8. (o) Relative abundance of 3-hydroxybutyric acid (3-HB) in cecal contents, *n* = 6–8. (p) Enrichment analysis of differential metabolite pathways, *n* = 6–8. (q) Peak area and quantitative maps of 3-HB content in the MRS Medium supernatant and *L. murinus* supernatant after 12 h anaerobic culture (MRS+*L. murinus*), *n* = 4. (r) 3-HB content in feces from WT, GF, and GF+*L. murinus* groups before surgery, *n* = 4. (s) 3-HB content in serum, *n* = 6–8. (t) Relative mRNA expression levels of Hmgcs2 in liver tissues, *n* = 6–8. The results are expressed as the median and quartile. * *p* < 0.05, ** *p* < 0.01, ***p < 0.001 by wetch’s t test (Q) and one-way ANOVA (Tukey’s test). GF: germ free; ND: normal diet; TRF: time-restricted feeding; ALT: alanine Aminotransferase; AST: aspartate aminotransferase; *L. murinus*: *lactobacillus murinus*; K*L. murinus*: killed *lactobacillus murinus*; Principal component analysis (PCA).

We transplanted preoperative fecal bacteria from ND and TRF mice into pseudo-GF mice (Figure S2a). The weights of the ND and TRF feces-FMT mice were similar (Figure S2b), and qPCR showed higher *L. murinus* abundance in TRF feces-FMT mice (Figure S2c), indicating successful FMT. TRF feces+CLP mice had improved 5-day survival, lower LPS levels, reduced liver damage and serum ALT, and AST levels compared to ND feces+CLP mice (Figures S2d-i). There were no significant differences in lung and kidney injury, except for lower creatinine levels in TRF feces+CLP mice (Figures S2j-n). Furthermore, in ABX-treated pseudo-GF mice, gut microbiota removal exacerbated septic liver injury and abolished the protective effect of TRF against septic injury (Figure S3). We then used GF mice to further validate the role of the gut microbiome, particularly the differential indicator species *L. murinus*, in the septic liver injury alleviated by TRF. Figure 2(a) shows the TRF patterns in GF mice. No significant difference was observed in the preoperative body weight between GF mice in the ND and TRF groups (Figure 2(b)). GF mice had higher serum LPS levels and more severe liver injury than WT mice after CLP, with or without TRF (Figure 2(c-g)). The protective effect of TRF against septic liver injury in WT mice was not observed in GF mice. However, *L. murinus* gavage significantly reduced serum LPS levels and liver injury in GF mice (Figure 2(c-g)). These results indicate that TRF alleviates septic liver injury by reshaping the gut microbiota, especially *L. murinus*

Subsequently, we further pretreated C57BL/6J mice with live or killed *L. murinus* via gavage for 1 week to establish a CLP model. Unlike killed *L. murinus*, pretreatment with live *L. murinus* decreased serum LPS, ALT, and AST levels and attenuated liver injury in the CLP model (Figure 2(h-l)). These results suggested that *L. murinus* attenuated septic liver injury via its active host metabolites. Additionally, *L. murinus* can effectively mitigate LPS-induced septic liver injury (Figure S4). Q200 targeted metabolomics analysis of cecal contents revealed significant differences in metabolite profiles among Sham, CLP, and CLP+*L. murinus* (CLPLM) groups, with metabolite 3-HB showing the greatest fold change (Figure S5a,b and Figure 2(m,n)). Compared to Sham mice, CLP mice had significantly reduced 3-HB levels, whereas live *L. murinus* treatment increased 3-HB levels (Figure 2(o)). Enrichment analysis identified ketone body metabolism as the most enriched pathway (Figures 2(p) and and S5c), prompting us to focus on 3-HB. LC/MS-MS experiments showed no significant difference in metabolite 3-HB content between *L. murinus* supernatant and MRS medium (Figure 2(q)), suggesting that *L. murinus* cannot directly produce the metabolite 3-HB. GF mice had reduced 3-HB levels in the feces compared with WT mice, but these levels increased after *L. murinus* treatment (Figure 2(r)). Additionally, 3-HB content in the cecal contents of CLP mice was lower than in Sham mice, and *L. murinus* treatment significantly increased 3-HB levels (Figure S5d), consistent with metabolomics results. These findings suggest that 3-HB may be a co-metabolite of *L. murinus* and its host. In addition, TRF elevated serum 3-HB levels in WT CLP mice, but in GF mice, 3-HB levels were reduced and unchanged between ND and TRF groups, though they increased with *L. murinus* treatment (Figure 2(s)). TRF feces+CLP mice had significantly higher serum 3-HB levels than ND feces+CLP mice (Figure 2(s)). In the meantime, the expression trend of hmgcs2, an essential enzyme for 3-HB synthesis, was consistent with that of 3-HB (Figure 2(t)). We also observed no differences in the abundance of *L. murinus* in the blood and liver tissues between the CLP and CLP+ *L. murinus* groups (Figure S5e). These results suggest that *L. murinus* may control 3-HB synthesis via Hmgcs2 regulation.

### *Hmgcs2 requirement in* L. murinus *for 3-HB synthesis to mitigate septic liver injury*

**Figure 3.**
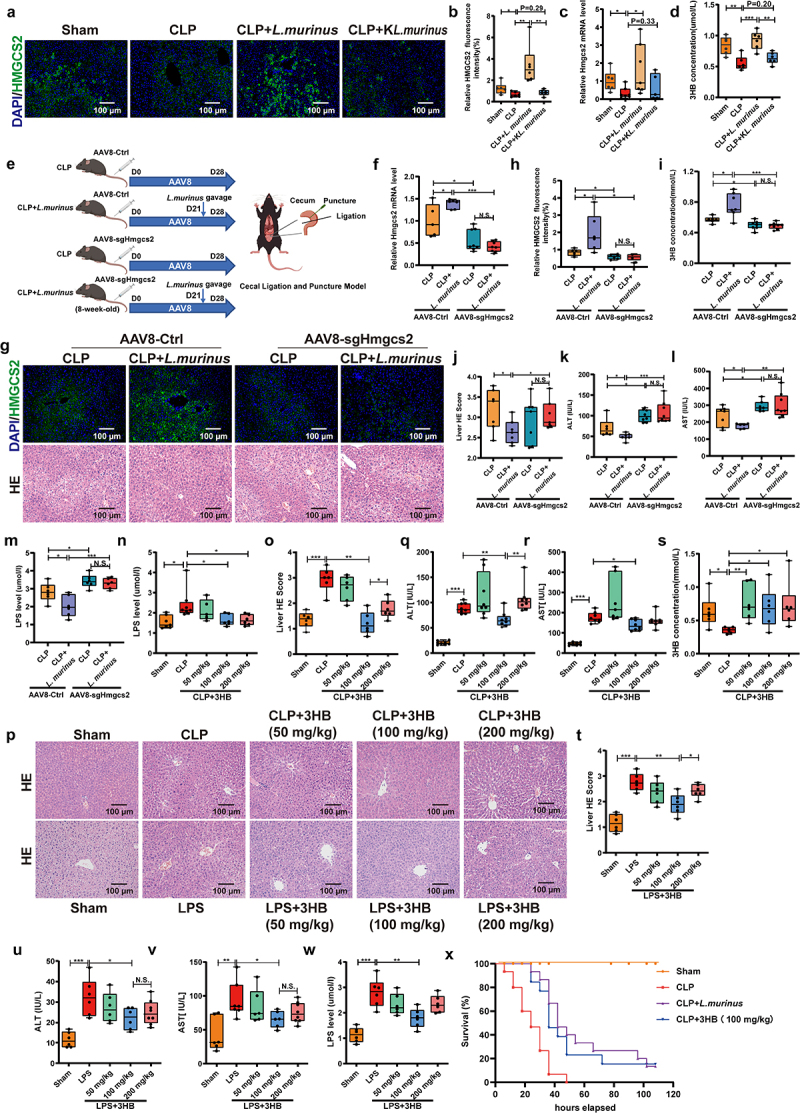
*Lactobacillus murinus* promotes 3-HB synthesis via the Hmgcs2 enzyme to alleviate septic liver injury. (a, b) HMGCS2 immunofluorescence in liver tissue from sham group, CLP group, CLP+*L. murinus* group and CLP+K*L. murinus* group and the quantification analysis, scale: 100 μm, *n* = 6. (c) Relative mRNA expression of Hmgcs2 in liver tissues of mice, *n* = 6–8. (d) Serum 3-HB content, *n* = 6–8. (e) Schematic diagram of Hmgcs2 gene knockout mice constructed by adeno-associated virus (AAV) technique. Hmgcs2 gene knockout AAV virus (AAV8-sgHmgcs2) and control AAV virus (AAV8-ctrl) were injected tail vein into WT C57BL/6J mice, respectively, and the CLP model was established 28 days later. At 21 days of AAV virus injection, mice in the CLP+*L. murinus* group were treated with *L. murinus* by gavage for 1 week. (f) Relative Hmgcs2 mRNA expression, *n* = 6–8. (g) HMGCS2 immunofluorescence and HE staining of liver tissue, scale: 100 μm, *n* = 6. (h) Quantitative analysis of HMGCS2 immunofluorescence intensity, *n* = 6. (i) Serum 3-HB content in mice, *n* = 6–8. (j) Liver histopathological damage score, *n* = 6. (k, l) Serum ALT and AST levels, *n* = 6–8. (m) LPS content in serum of mice, *n* = 6–8. (n) Serum LPS in sham, CLP, CLP + 3-HB (50, 100, 200 mg/kg) groups, *n* = 6–8. (o, p) HE staining in the liver tissues and quantification, scale: 100 μm, *n* = 6. (q-s) Serum levels of ALT, AST and 3-HB in CLP model mice, *n* = 6–8. (t) Liver histopathological damage score in LPS model mice, *n* = 6. (u-w) ALT, AST and LPS levels in serum of LPS model mice, *n* = 6–8. (x) 5-day survival in sham, CLP, CLP+ *L. murinus* and CLP + 3-HB (100 mg/kg) groups (*n* = 20). The results are expressed as the median and quartile. * *p* < 0.05, ** *p* < 0.01, *** *p* < 0.001 by two -way ANOVA (Tukey’s test) (F, H-M), one-way ANOVA (Tukey’s test) (B-D, N-W) and log-rank test (X). AAV: adeno-associated Virus; 3-HB: 3- hydroxybutyric acid; ALT: alanine Aminotransferase; AST: aspartate aminotransferase; *L. murinus*: *lactobacillus murinus*; K*L. murinus*: killed *lactobacillus murinus.*

We used Hmgcs2 knockout mice to investigate whether *L. murinus* could alleviate septic liver injury by promoting 3-HB synthesis via Hmgcs2. Immunofluorescence and mRNA analysis showed lower HMGCS2 expression in CLP mice liver tissue compared with that in Sham mice, with pre-treatment of live *L. murinus* upregulating HMGCS2 expression in CLP mice, but not K*L.murinus* (Figure 3(a-c)). Additionally, live *L. murinus* also increased the serum 3-HB levels in CLP mice, whereas K*L. murinus* had no such effect (Figure 3(d)). We then injected Hmgcs2 knockout AAV virus (AAV8-sgHmgcs2) and Control AAV virus (AAV8-Ctrl) into WT mice, followed by CLP after 28 days (Figure 3(e)). Hmgcs2 knockout (AAV8-sgHmgcs2) reduced HMGCS2 expression and serum 3-HB levels, which were restored by *L. murinus* in AAV8-Ctrl but not in knockout mice (Figure 3(f–i)). In AAV8-Ctrl mice, *L. murinus* pre-treatment reduced LPS, ALT, and AST levels and attenuated liver histopathological damage in CLP mice (Figure 3(g,j-m)). However, in AAV8-sgHmgcs2 mice, *L. murinus* pre-treatment failed to exert these protective effects (Figure 3(g,j-m)). The Hmgcs2 inhibitor, hymeglusin, also reversed the attenuation of septic liver injury by *L. murinus* (Figures S6a-e). Additionally, Hmgcs2 overexpression reduced LPS-induced toxicity compared to AAV-Ctrl transfected cells (Figures S6f,g). These results suggest that Hmgcs2 knockout exacerbates septic injury in mice and *L. murinus* requires Hmgcs2 for 3-HB synthesis to alleviate septic liver injury.

Pre-treatment with the 100 mg/kg 3-HB sodium salt significantly decreased serum LPS, ALT, and AST levels in CLP mice and attenuated morphological liver injury (Figure 3(n-r)). Simultaneously, this pre-treatment also increased serum 3-HB levels in CLP mice (Figure 3(s)). Similarly, in the LPS-induced sepsis model, 3-HB sodium salt pre-treatment effectively attenuated septic liver injury and reduced serum LPS, ALT, and AST levels (Figure 3(p,t-w)). In addition, 3-HB sodium salt and *L. murinus* significantly increased the 5-day survival rate of CLP mice (Figure 3(x)). These results suggest that the 3-HB sodium salt has a protective effect against septic liver injury.

## Analysis of the correlation between serum 3-HB levels and liver injury marker in patients with septic liver injury

**Figure 4.**
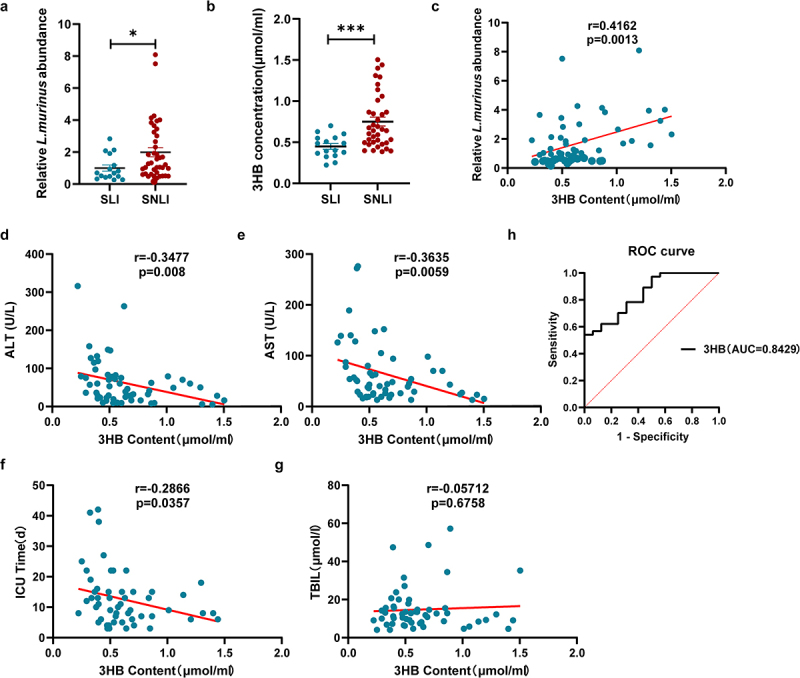
Analysis of the correlation between serum 3-HB levels and liver injury marker in patients with septic liver injury. (a) Fecal *L. murinus* abundance in patients with sepsis with or without liver injury, SLI (*n* = 17), SNLI (*n* = 40). (b) Serum 3-HB levels in patients with sepsis with or without liver injury, SLI (*n* = 17), SNLI (*n* = 40). (c) Correlation analysis of serum 3-HB content with *L. murinus* abundance in patients with sepsis with or without liver injury, respectively, *n* = 57. (d-g) correlation analysis of serum 3-HB content with serum ALT level, AST level, ICU stay, and total bilirubin level in patients with sepsis with or without liver injury, respectively, *n* = 57. (h) ROC curve analysis. *** *p* < 0.001 by wetch’s t test (a, b) spearman analysis in (c-g).

This study enrolled 57 patients with sepsis, including 17 patients in the sepsis liver injury (SLI) group and 40 patients in the sepsis non-liver injury (SNLI) group. Baseline characteristics, such as gender, age, and SOFA scores, were similar between the groups (Table S2). However, the serum levels of lactate, ALT, AST, and ICU stay time were significantly higher in the patients in the SLI group than those in the SNLI group. *L. murinus* abundance was quantified by qPCR in fecal samples from patients with SLI and SNLI groups (Figure 4(a)). Serum 3-HB levels were significantly lower in patients in the SLI group compared with the SNLI group (Figure 4(b)). Figure 4(c) shows a significant positive correlation between serum 3-HB level and *L. murinus* abundance in patients. Furthermore, correlation analysis revealed a negative association between serum 3-HB levels and ALT, AST levels, and ICU stay time in both groups, but no correlation was observed with total bilirubin levels (Figure 4(d-g)). ROC curve analysis indicated that serum 3-HB levels had the potential to predict liver injury in patients with sepsis, with an AUC value of 0.8429 (Figure 4(h)).

### 3-HB activates the PI3K/AKT/mTOR/LPIN1 signaling pathway to protect against septic liver injury

**Figure 5.**
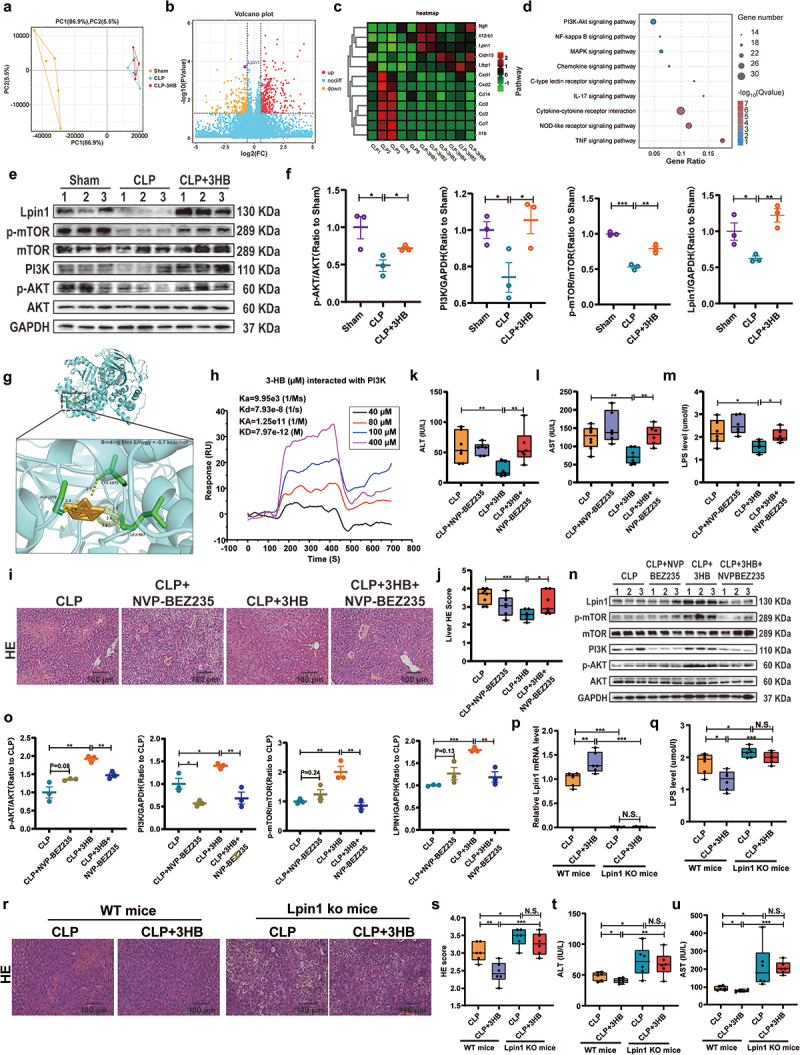
3-HB activates the PI3K/AKT/mTOR/LPIN1 signaling pathway to protect against septic liver injury. (a) Principal component analysis, *n* = 5–6. (b) Volcanic plot of differential genes between CLP and CLP + 3-HB groups. (c) Differential gene heat map of CLP and CLP + 3-HB groups. (d) KEGG pathway analysis. (e, f) protein expression levels of AKT, p-akt, PI3K, mTOR, p-mTOR and LPIN1 in liver tissue were detected by WB (*n* = 3). (g) Molecular docking analysis of 3-HB with the PI3K protein (PDB: 1E8Y). (h) SPR analysis of 3-HB (40, 80, 100 and 400 μM) binding to the recombinant human (rh) protein of rhPIK3R3 (Sf9, his, GST). (i) HE staining of liver tissues from mice in the CLP, CLP+NVP-BEZ235, CLP + 3-HB, and CLP + 3-HB+NVP-BEZ235 groups, scale: 100 μm, *n* = 6. (j) Liver histopathological damage scores, *n* = 6. (k, l) ALT and AST content in serum from mice in the CLP, CLP+NVP-BEZ235, CLP + 3-HB, and CLP + 3-HB+NVP-BEZ235 groups, *n* = 6–8. (m) LPS content in serum from mice in the CLP, CLP+NVP-BEZ235, CLP + 3-HB, and CLP + 3-HB+NVP-BEZ235 groups, *n* = 6–8. (n, o) the protein expression levels of p-akt, PI3K, p-mTOR and LPIN1 in liver tissue (*n* = 3). (p) Relative Lpin1 mRNA expression level, *n* = 6. (q) LPS content in serum from WT and Lpin1KO mice, *n* = 6. (r, s) HE staining and liver histopathological damage scores, scale: 100 μm, *n* = 6. (t, u) ALT and AST content in serum from WT and Lpin1KO mice, *n* = 6–8. The results are expressed as the mean ± SEM (F, O) and the median and quartile. * *p* < 0.05, ** *p* < 0.01, *** *p* < 0.001 by one-way ANOVA (Tukey’s test)(f) and two-way ANOVA (Tukey’s test)(K-U). WB: Western blot; 3-HB: 3-hydroxybutyric acid; ALT: alanine Aminotransferase; AST: aspartate aminotransferase; MDA: Malondialdehyde; LPO: lipid Peroxidation; PCA: Principal component Analysis; SEM, standard error of mean; rh: recombinant human.

In this study, transcriptome sequencing analysis of liver tissues from Sham, CLP, and CLP + 3-HB mice was conducted to explore how 3-HB alleviates septic liver injury. PCA showed distinct differences among groups (Figure 5(a)). Differential gene analysis showed that Lpin1 was significantly upregulated in the CLP + 3-HB group compared to that in the CLP group (Figure 5(b,c)), which was further confirmed by qPCR (Figure S7a). KEGG analysis indicated that the PI3K/AKT pathway was significantly enriched in the CLP + 3-HB group (Figure 5(d)). Western blot (WB) confirmed that 3-HB modulates the PI3K/AKT/mTOR/LPIN1 pathway. Compared with Sham group mice, CLP group mice significantly inhibited phosphorylation of AKT (p-AKT/AKT) and mTOR (p-mTOR/mTOR), and decreased PI3K and LPIN1 protein expression, which were significantly improved after 3-HB pretreatment (Figure 5(e,f)). Molecular simulations with PI3K (PDB: 3FXI) revealed that 3-HB forms a stable complex with PI3K (binding free energy = −5.7 kcal/mol) (Figure 5(g)). Meanwhile, SPR analysis further indicated that 3-HB binds rapidly to recombinant human PI3K (rhPIK3R3) (Sf9, His, GST) with an equilibrium dissociation constant (K_D_) of 7.97 × 10^− 1 2^ M (Figure 5(h)). These results revealed that 3-HB directly targeted PI3K.

NVP-BEZ235, a dual inhibitor of the PI3K/AKT/MTOR pathway, was utilized to investigate whether 3-HB attenuated septic liver injury by activating the PI3K/AKT/MTOR pathway. HE staining and biochemical indices revealed that 3-HB effectively reduced CLP-induced liver damage and serum LPS, ALT, and AST levels, whereas the inhibitor NVP-BEZ235 reversed these effects (Figure 5(i-m)). WB analyses showed that 3-HB increased p-AKT/AKT, p-mTOR/mTOR, PI3K and LPIN1 protein expression levels in the liver compared to CLP alone, while NVP-BEZ235 inhibited these effects (Figure 5(n,o))

Lpin1, a downstream target of the PI3K/AKT/mTOR pathway. Lpin1 KO mice were used to investigate whether 3-HB attenuated septic liver injury by activating the Lpin1 pathway. In WT mice, Lpin1 mRNA expression in the liver tissue was significantly higher in the CLP + 3-HB group than that in the CLP group, whereas Lpin1 KO mice significantly decreased Lpin1 expression (Figure 5(p)). In WT mice, 3-HB administration significantly decreased serum LPS levels and liver injury of CLP mice; however, these effects were absent in Lpin1 KO mice (Figure 5(q-u)). Additionally, Lpin1 KO mice showed exacerbated septic liver injury after CLP (Figure 5(q-u)). Lpin1 KO mice showed elevated *Il-1β*, *Il-6*, and *Tnf-α* in liver tissue, with inhibited 3-HB-mediated downregulation of these factors (Figure S7b). These results suggest that Lpin1 signaling plays a critical role in the attenuation of septic liver injury by 3-HB. Collectively, these findings suggest that 3-HB pre-treatment alleviates septic liver injury by activating the PI3K/AKT/mTOR/LPIN1 signaling pathway.

### 3-HB inhibits ferroptosis in mice by activating the PI3K/AKT/mTOR/LPIN1 pathway

**Figure 6.**
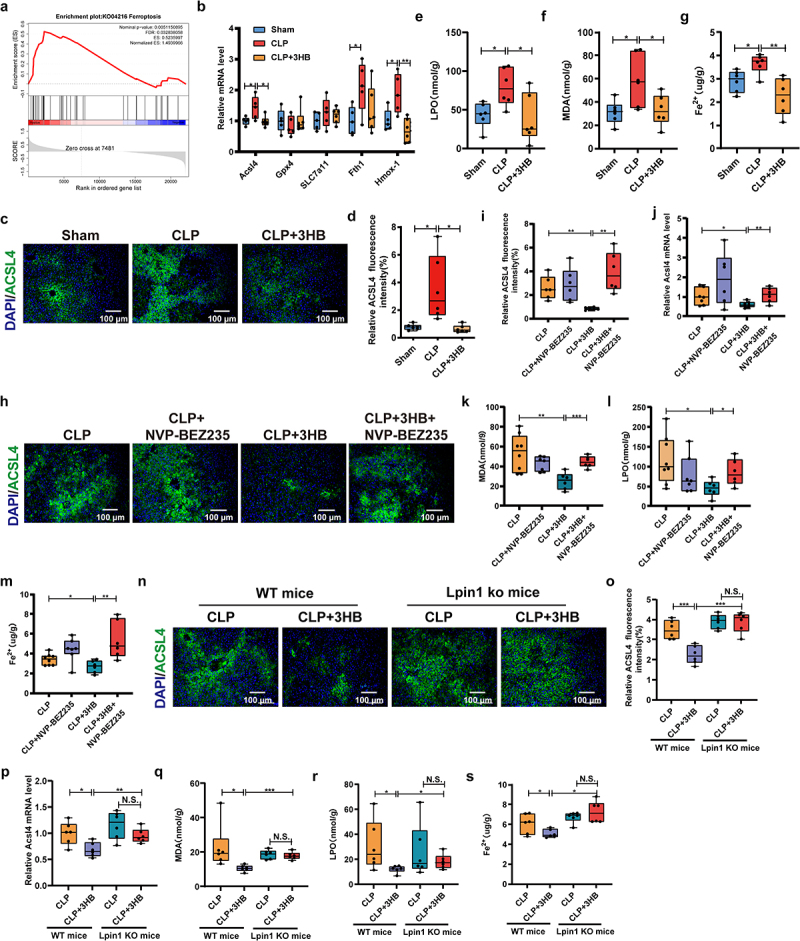
3-HB inhibits ferroptosis in mice by activating the PI3K/AKT/mTOR/LPIN1 pathway. (a) GSEA-kegg analysis. (b) Relative mRNA levels of Acsl4, Gpx4, SLC7a11, Fth1 and hmox-1 genes, *n* = 5–6. (c, d) ACSL4 immunofluorescence and quantification in liver tissues, scale: 100 μm, *n* = 6. (e-g) MDA, LPO and Fe^2+^ content in liver tissue, *n* = 6. (h, i) ACSL4 immunofluorescence and quantification in liver tissues, scale: 100 μm, *n* = 6. (j) Relative ACSL4 mRNA expression level, *n* = 6–8. (k-m) MDA, LPO and Fe^2+^ content in liver tissue from mice in the CLP, CLP+NVP-BEZ235, CLP + 3-HB, and CLP + 3-HB+NVP-BEZ235 groups, *n* = 6. (n) ACSL4 immunofluorescence of liver tissue from WT and Lpin1KO mice, scale: 100 μm, *n* = 6. (o) Quantitative analysis of ACSL4 immunofluorescence intensity, *n* = 6. (p) Relative ACSL4 mRNA expression level, *n* = 6–8. (q-s) MDA, LPO and Fe^2+^ content in liver tissue from WT and Lpin1KO mice, *n* = 6. The results are expressed as the median and quartile. * *p* < 0.05，** *p* < 0.01，*** *p* < 0.001 by one-way ANOVA (Tukey’s test)(B-G) and two-way ANOVA (Tukey’s test). 3-HB: 3-hydroxybutyric acid; ALT: alanine Aminotransferase; AST: aspartate aminotransferase; MDA: Malondialdehyde; LPO: lipid Peroxidation; SEM, standard error of mean.

GSEA-KEGG analysis revealed that the ferroptosis pathway was significantly enriched in the CLP group (Figure 6(a)). Ferroptosis-related genes (*Acsl4*, *Fth1*, *Hmox-1*) were upregulated in the CLP group and downregulated after 3-HB treatment, with no significant changes in *Gpx4* and *SLC7a11* (Figures S7c and 6(b) and 6(b)). Consistent with this, GPX4 protein levels showed no significant differences among the NC, LPS, and LPS + 3-HB-treated AML12 cells (Figures S7d). Further analysis of ferroptosis-related markers revealed that 3-HB pretreatment significantly reduced ACSL4 protein expression in CLP mice (Figure 6(c,d)) and decreased MDA, LPO, and Fe^2+^ levels in the liver tissue (Figure 6(e-g)). The PI3K/AKT/mTOR pathway plays a crucial role in regulating ferroptosis by modulating lipid metabolism and antioxidant capacity. As shown in Figure 6(h-j), the CLP + 3-HB group significantly downregulated the protein and mRNA expression of ACSL4 compared with the CLP group, whereas the CLP + 3-HB+NVP-BEZ235 group upregulated ACSL4 expression. Meanwhile, MDA, LPO, and Fe^2+^ levels in the liver tissue of mice in the CLP + 3-HB group were significantly lower than those in the CLP group; however, NVP-BEZ235 notably increased these ferroptosis-related indicators (Figure 6(k-m)).

Bioinformatics analysis identified *Lpin1* as a ferroptosis-related gene and a potential biomarker for ferroptosis. In WT mice, 3-HB lowered ACSL4 protein levels and reduced MDA, LPO, and Fe^2+^ levels, but these effects were not observed in Lpin1 KO mice (Figure 6(n-s)). Furthermore, Lpin1 KO mice in the CLP + 3-HB group had higher ACSL4 expression and MDA, LPO, and Fe^2+^ levels compared to WT mice (Figure 6(n-s)). Lpin1 knockout mice generated by AAV8 also confirmed that lpin1 knockout reversed the protective effect of 3-HB against septic liver injury and ferroptosis inhibition (Figures S7e-p). As shown in Figures S8a-d, HE staining and biochemical markers (ALT, AST) showed that Fer, a ferroptosis inhibitor significantly alleviated septic liver injury in CLP mice. In contrast, NVP-BEZ235 exacerbated liver injury, but this effect was reversed by Fer. Ferroptosis-related markers further confirmed that mTOR mitigates septic liver injury by inhibiting ferroptosis (Figures S8e-g). In summary, these studies reveal that 3-HB alleviates septic liver injury in mice by activating the PI3K/AKT/mTOR/LPIN1 pathway to inhibit ferroptosis.

### 3-HB alleviates AML12 cell injury by activating the PI3K/AKT/mTOR/LPIN1 pathway to inhibit ferroptosis

**Figure 7.**
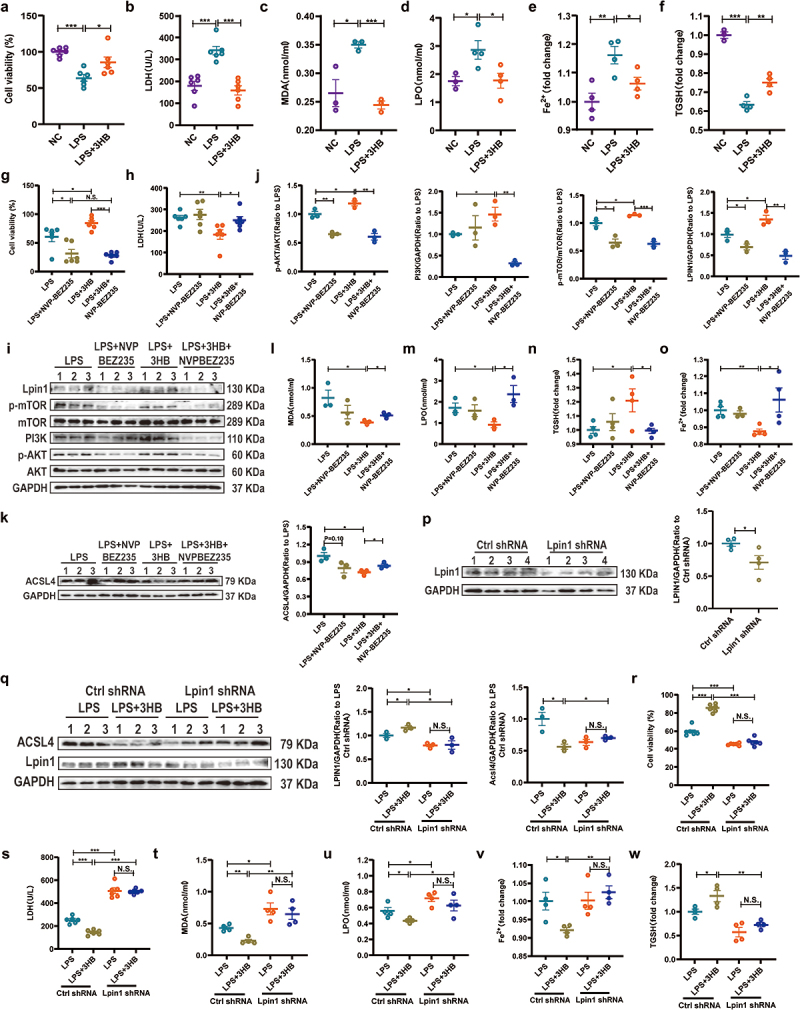
3-HB alleviates AML12 cell injury by activating the PI3K/AKT/mTOR/LPIN1 pathway to inhibit ferroptosis. (a) AML12 cell viability was detected by CCk8, *n* = 6. (b) LDH content in AML12 cell supernatant, *n* = 6. (c-f) MDA, LPO, TGSH and Fe^2+^ content in the AML12 cells, *n* = 4. (g) AML12 cell viability by CCk8, *n* = 6. (h) LDH content in AML12 cell supernatant, *n* = 6. (i-k) the protein expression levels of p-akt, PI3K, p-mTOR, LPIN1 and ACSL4 in the AML12 cells treated with LPS, LPS+NVP-BEZ235, LPS + 3-HB, and LPS + 3-HB+NVP-BEZ235 (*n* = 3). (l-o) MDA, LPO, TGSH and Fe^2+^ content in the AML12 cells treated with LPS, LPS+NVP-BEZ235, LPS + 3-HB, and LPS + 3-HB+NVP-BEZ235, *n* = 4. (p) LPIN1 protein expression level, *n* = 4. (q) The protein expression levels of LPIN1 and ACSL4 in the AML12 cells (*n* = 3). (r) AML12 cell viability by CCk8, *n* = 6. (s) LDH content in AML12 cell supernatant, *n* = 6. (t-w) MDA, LPO, TGSH and Fe^2+^ content in AML12 cells transfected with Ctrl shRNA and Lpin1 shRNA, *n* = 4. The results are expressed as the mean ± SEM. *p < 0.05，**p < 0.01，***p < 0.001 by one-way ANOVA (Tukey’s test) (A-F) and two-way ANOVA (Tukey’s test). 3-HB: 3-hydroxybutyric acid; MDA: Malondialdehyde; LPO: lipid Peroxidation; SEM, standard error of mean.

*In vitro* validation of hepatocytes was also performed. 3-HB improved cell viability and reduced LDH content in LPS-stimulated AML12 cells (Figure 7(a,b)). WB analysis showed that LPS inhibited AKT and mTOR protein phosphorylation, downregulated PI3K and LPIN1 protein expression, and upregulated ACSL4 protein expression in AML12 cells (Figures S9a-f). Nevertheless, 3-HB pre-treatment reversed these changes, indicating a protective effect against LPS-induced cellular stress (Figures S9a-f). Furthermore, LPS-treated AML12 cells exhibited increased MDA, LPO, and Fe^2+^ levels compared to those in the NC group, whereas 3-HB pre-treatment reduced these levels (Figure 7(c-e)). TGSH levels were significantly reduced in the LPS group compared to both the NC and LPS + 3-HB groups (Figure 7f). Collectively, these findings suggest that 3-HB pre-treatment could affect hepatocyte ferroptosis.

CCK8 and LDH experiments showed that 3-HB significantly enhanced AML12 cell viability compared to the LPS group, but this effect was significantly reversed by NVP-BEZ235 in the LPS + 3-HB+NVP-BEZ235 group (Figure 7(g,h)). 3-HB treatment upregulated PI3K/AKT/mTOR/LPIN1 pathway proteins and downregulated ACSL4 in AML12 cells, whereas NVP-BEZ235 negated this effect (Figures 7(i-k)). Furthermore, The LPS + 3-HB group also showed decreased MDA, LPO, and Fe^2+^ levels and increased TGSH content compared to the LPS group; these effects were reversed by NVP-BEZ235 (Figures 7(l-o)). *In vitro*, Lpin1 shRNA reduced LPIN1 expression in AML12 cells (Figure 7(p)), and while 3-HB increased LPIN1 levels and decreased ACSL4 expression in Ctrl shRNA-transfected cells, these effects were lost with Lpin1 shRNA (Figure 7(q)). Lpin1 shRNA transfection also significantly reduced cell viability and promoted hepatocyte ferroptosis compared to Ctrl shRNA-transfected cells. The protective effects of 3-HB against LPS-induced hepatocyte injury and ferroptosis were inhibited by Lpin1 knockdown (Figure 7(r-w)). Additionally, Figures S9g-j shows that NVP-BEZ235 compromises cell viability by promoting ferroptosis, an effect counteracted by Fer. In summary, these studies reveal that 3-HB alleviates AML12 cell injury by activating the PI3K/AKT/mTOR/LPIN1 pathway to inhibit ferroptosis.

## Discussion

Sepsis-induced liver injury seriously affects patient prognosis, yet effective treatment remains unclear. This study showed that TRF can reduce septic liver injury by modulating the gut microbiota, particularly by increasing *L. murinus* abundance. Moreover, it was found that live *L. murinus* could markedly elevate serum levels of metabolite 3-HB and alleviate sepsis-related injury, while the knockout of the key enzyme for 3-HB synthesis Hmgcs2 in the liver negated this protective effect. More strikingly, clinical evidence has revealed that serum 3-HB levels are significantly positively correlated with *L. murinus* abundance and negatively correlated with liver injury indicators in septic patients. Serum 3-HB levels were found to predict septic liver injury with an AUC of 0.8429, indicating 3-HB as a potential predictor of septic liver injury. Additionally, we for the first time revealed that metabolite 3-HB activates the PI3K/AKT/MTOR/LPIN1 signaling pathway to inhibit hepatocyte ferroptosis, thereby alleviating septic liver injury. This study demonstrates that TRF reduces septic liver injury by modulating gut microbiota to increase *L. murinus*, which elevates 3-HB to activate PI3K/AKT/mTOR/LPIN1 and inhibit hepatocyte ferroptosis. In summary, this study elucidates the protective mechanism of TRF against septic liver injury and identifies 3-HB as a potential therapeutic target and predictive biomarker, thereby providing new insights into the clinical management and diagnosis of septic liver injury.

This study demonstrates that TRF exhibits a new potential in treating sepsis by improving metabolic homeostasis and modulating gut microbiota, specifically by increasing *L. murinus* levels. We found that live *L. murinus* effectively reduced septic liver injury, unlike inactivated bacteria. Similarly, live AAK bacteria mitigated organ damage in sepsis through its metabolite (novel tripeptide RKH).^11^ LC/MS-MS experiments revealed that 3-HB to be a co-metabolite of *L. murinus* and its host. The enzyme Hmgcs2, a key rate-limiting enzyme for 3-HB synthesis, plays a crucial role in various health conditions such as diabetes, tumors, fatty liver, and Alzheimer’s disease.^43–45^ Studies have shown that upregulation of Hmgcs2 in the liver supports intestinal cell differentiation and homeostasis maintenance, which are hindered by its downregulation.^46^ In addition, other study showed that colonization with specific butyric acid-producing bacteria was associated with increased 3-HB levels and ameliorated cardiac damage in Hmgcs2 KO mice, particularly in those lacking endogenous ketone body production.^47^ However, the present study on Hmgcs2 knockout mice revealed that *L. murinus* colonization did not confer protection against septic liver damage, suggesting that *L. murinus* relies on endogenous ketone body metabolism to exert its beneficial effects. Additionally, we observed no differences in the abundance of *L. murinus* in blood and liver tissues between the CLP and CLP+*L. murinus* groups. This suggests that *L. murinus* might affect liver Hmgcs2 enzyme activity through alternative pathways in the gut, thereby promoting 3-HB synthesis. It is known that the gut microbiota influences liver health and disease through the gut-liver axis. Metabolites such as trimethylamine N-oxide (TMAO) and free fatty acids, produced by the gut microbiota, reach the liver via the portal vein, regulating bile acid synthesis, glucose, and lipid metabolism, and impacting liver physiology.^48,49^ Despite our experimental results showing that *L. murinus* may not have directly entered the liver, this does not entirely rule out the possibility that bacterial components or metabolites could influence the liver via the circulatory system. Furthermore, in GF mouse experiments, *L. murinus* administration increased Hmgcs2 expression in liver tissues, suggesting a possible direct or indirect interaction between the bacterium and liver metabolism. Given that *L. murinus* does not directly produce 3-HB, other metabolites or mechanisms may be involved. Moreover, *L. murinus* may collaborate with other intestinal bacteria to produce metabolites affecting Hmgcs2. The present study acknowledges a limitation in understanding how *L. murinus* influences host 3-HB production through the Hmgcs2 enzyme in the liver, thereby warranting further exploration of the complex mechanisms and potential pathways involved.

Recent studies have shown that 3-HB sodium salt supplementation can effectively reduce sepsis-induced lung and kidney injuries, myasthenia, and cognitive dysfunction.^42,50,51^ This research highlights its novel protective effects against liver injury in sepsis mouse models induced by CLP and LPS. However, high concentrations may risk ketoacidosis, and excessive sodium intake can lead to complications such as cerebrovascular and renal dysfunction.^52^ Our study demonstrates that the lower dose of 3-HB (100 mg/kg) is more effective in protecting liver function in sepsis models compared to the higher dose (200 mg/kg). This aligns with findings from Zhang et al., who reported that 100 mg/kg 3-HB significantly reduced systemic inflammation in atherosclerosis mice, while 200 mg/kg was ineffective.^53^ Studies on GPCR activation demonstrate that different concentrations of ligands can induce varying GPCR activation modes, leading to distinct responses.^36^ This may explain the reduced efficacy of higher 3-HB concentrations. Furthermore, similarly, Weckx et al. identified a toxic threshold for 3-HB in sepsis models, with 150 mg/day prevented muscle weakness without toxicity, but higher doses increased disease severity and mortality,^54^ suggesting potential dose-dependent toxicity. These findings underscore the importance of optimizing 3-HB dosing and further investigating its concentration-dependent effects and potential cytotoxicity. In the present study, serum 3-HB levels were significantly reduced in patients with septic liver injury, with an AUC of 0.8429 for predicting such injuries, suggesting 3-HB as a potential biomarker for predicting septic liver injury. In patients with septic liver injury, liver function impairment may disrupt energy metabolism. Supplementation with 3-HB sodium salt can provide an alternative energy source and help maintain ketone body balance. However, treatment should be individualized based on patient conditions and healthcare provider recommendations. Sepsis patients often have impaired gut function. TRF may affect gut microbiota, impacting nutrition stability and gut recovery. Gut dysfunction exacerbates malnutrition and inflammation, prolonging recovery. Early enteral nutrition improves nutritional status, shortens ICU stays, and reduces complications.^55^ This suggests that a reasonable dietary pattern, including TRF may positively impact nutritional management and clinical outcomes in sepsis. Despite limited direct studies on TRF in sepsis, our findings and recent advances in sepsis nutrition provide a theoretical basis for its application. Future research should assess TRF’s effects on gut function and clinical outcomes in sepsis patients to verify its safety and efficacy.

The development of sepsis involves an imbalance between pro- and anti-inflammatory responses, with the PI3K/AKT/mTOR pathway as a crucial inflammatory response. Research has demonstrated that sivelestat enhances myocardial dysfunction by activating this pathway,^56^ while dexmedetomidine can mitigate acute kidney injury by inhibiting this pathway and promoting autophagy.^25^ The complex role of this pathway in sepsis warrants further investigation. This study validated that 3-HB activates the PI3K/AKT/mTOR pathway through SPRI molecular docking experiments, as well as *in vitro* and *in vivo* studies. Lpin1, a key player in lipid synthesis and metabolism, is a downstream target of the PI3K/AKT/mTOR signaling pathway^29^ and is implicated in sepsis immune response, inflammation, and ferroptosis.^30,57^ Research suggests that 3-HB selectively influences Lpin1 mRNA expression during the regulation of milk fat synthesis in bovine mammary epithelial cells.^58^ While the specific role of Lpin1 in septic injury remains unclear, this study utilized Lpin1 shRNA, AAV8-Lpin1sgRNA, and Lpin1 KO mice to demonstrate that Lpin1 deficiency exacerbated septic liver damage in CLP mice and abolished the protective effects of 3-HB, including the reduction of ACSL4 expression and the decrease in MDA, LPO, and Fe^2 +^ levels in both liver tissues and AML12 cells. These findings collectively highlight the critical role of lpin1 in mediating the protective effects of 3-HB, likely through its regulation of lipid metabolism and ferroptosis-related pathways. While Zhou et al. reported that adipose-specific lipin-1 overexpression exacerbates hepatic ferroptosis,^32^ our findings demonstrate a hepatoprotective role of lipin-1 activation in sepsis. Furthermore, Lpin1 deficiency has been shown to aggravate impaired fatty acid β-oxidation and renal damage in diabetic nephropathy patients.^59^ This discrepancy underscores the importance of tissue-specific and disease context in lipin-1 signaling. Under septic conditions, hepatic lipin-1 activation reduced ACSL4 expression and lipid peroxidation, thereby counteracting ferroptosis. Future studies should explore the dual roles of lipin-1 in systemic versus localized metabolic regulation.

Sepsis pathogenesis involves various cell death mechanisms, including apoptosis, necroptosis, pyroptosis, autophagy, and ferroptosis. This study focused on ferroptosis, given its significant enrichment in the CLP group compared with the CLP + 3-HB group. Recent studies have shown that the PI3K/AKT/mTOR pathway is closely related to ferroptosis. Inhibition of PI3K, AKT, or mTOR can enhance the sensitivity of cancer cells to ferroptosis, primarily by reducing the expression of sterol regulatory element-binding protein 1 (SREBP1), a core transcription factor regulating lipid metabolism.^60^ SLC38A5 suppresses ferroptosis through glutamine-mediated activation of the PI3K/AKT/mTOR signaling in osteosarcoma.^61^ Existing literature suggests that targeting ferroptosis could be a novel therapeutic approach to mitigate septic liver damage.^62,63^ Pre-treatment with 3-HB reduced ferroptosis by downregulating the ferroptosis-related factor ACSL4 in CLP mice, reducing levels of MDA, LPO, and Fe^2+^ in the liver tissue. Additionally, Fer significantly alleviated septic liver injury, while NVP-BEZ235 exacerbated it, an effect reversed by Fer. These results indicate that mTOR mitigates septic liver injury by inhibiting ferroptosis. The protective effects of 3-HB are counteracted by NVP-BEZ235, which blocks the PI3K/AKT/mTOR pathway and promotes ferroptosis. These findings highlight the critical role of the PI3K/AKT/mTOR/LPIN1 axis in regulating ferroptosis and suggest potential therapeutic strategies for septic liver injury. ACSL4, a key enzyme in lipid peroxidation-induced ferroptosis,^64^ exacerbates LPS-induced mortality in a septic shock model when deficient.^65^ Conversely, SHP2 knockout mitigates LPS-induced ferroptosis by suppressing ACSL4 expression in acute lung injury.^66^ Vitamin D has also demonstrated a protective effect against ulcerative colitis by inhibiting ACSL4-mediated ferroptosis.^67^ The role of ACSL4 in septic injury-related ferroptosis is complex and warrants further investigation. Ferroptosis in septic liver injury extends beyond hepatocytes to include macrophages and neutrophils, emphasizing the need for additional research. The precise mechanism of action of 3-HB in septic liver injury remains complex and ambiguous, with this study only scratching the surface of the potential mechanisms. A deeper understanding of these processes may lead to new strategies for sepsis management.

Our study is not without limitations. While we have examined the impact of TRF on sepsis regulation through *L. murinus* and 3-HB, several unexplored areas warrant further investigation. Other bacteria and metabolites might also influence sepsis regulation and protective effects. Although, we observed no differences in the abundance of *L. murinus* in blood and liver tissues between the CLP and CLP+*L. murinus* groups, the precise mechanism by which *L. murinus* influences the Hmgcs2 enzyme to modulate 3-HB production in the host remains unknown, as does the complex role of 3-HB in septic liver injury. Moreover, clinical data on TRF intervention and gut microbiota is scarce, limiting clinical application. Future research will further explore these aspects to fill in the gaps and provide new insights and strategies for the treatment of septic patients. This study demonstrates that TRF reduces septic liver injury by modulating gut microbiota to increase *L. murinus*, which elevates 3-HB to activate PI3K/AKT/mTOR/LPIN1 and inhibit hepatocyte ferroptosis. In summary, this study elucidates the protective mechanism of TRF against septic liver injury and identifies 3-HB as a potential therapeutic target and predictive biomarker, thereby providing new insights into the clinical management and diagnosis of septic liver injury.

## List of abbreviations

**Table:** 

3-HB	3-hydroxybutyrate
AAV	Adeno-Associated Virus
ABX	antibiotic
ALT	alanine aminotransferase
AST	aspartate aminotransferase
BUN	Blood Urea Nitrogen
CLP	Cecal ligation and puncture
Fer	ferrostatin-1
FMT	fecal microbiota transplantation
GF	germ-free
HE	hematoxylin-eosin; Hmgcs2
3-hydroxy	3-methylglutaryl-CoA synthase 2
LDH	lactate dehydrogenase
*L. murinus*	*Lactobacillus murinus*
LPO	lipid peroxide
LPS	lipopolysaccharide
MDA	malondialdehyde
ND	normal diet
OPLS-DA	Orthogonal partial least square discriminant analysis
OTUs	operational taxonomic units
PAMPs	pathogen-associated molecular pattern
rh	recombinant human
SEM	standard error of mean
SLI	sepsis liver injury
SNLI	sepsis non-liver injury
T-GSH/GSSG	total glutathione/oxidized glutathione
TRF	time-restricted feeding.

## Supplementary Material

Supplemental Material

## Data Availability

The raw sequencing data generated from this study have been deposited in NCBI Sequence Read Archive (SRA) (http://www.ncbi.nim.nih.gov/sra) under the accession number PRJNA1132404. All other data associated with this study are present in the paper or Supplementary Materials.

## References

[1] Markwart R, Saito H, Harder T, Tomczyk S, Cassini A, Fleischmann-Struzek C, Reichert F, Eckmanns T, Allegranzi B. Epidemiology and burden of sepsis acquired in hospitals and intensive care units: a systematic review and meta-analysis. Intensive Care Med. 2020;46(8):1536–28. doi: 10.1007/s00134-020-06106-2. 32591853 PMC7381455

[2] Hollenberg SM, Singer M. Pathophysiology of sepsis-induced cardiomyopathy. Nat Rev Cardiol. 2021;18(6):424–434. doi: 10.1038/s41569-020-00492-2. 33473203

[3] Singer M, Deutschman CS, Seymour CW, Shankar-Hari M, Annane D, Bauer M, Bellomo R, Bernard GR, Chiche J-D, Coopersmith CM, et al. The third international consensus definitions for sepsis and septic shock (sepsis-3). Jama. 2016;315(8):801–810. doi: 10.1001/jama.2016.0287. 26903338 PMC4968574

[4] Strnad P, Tacke F, Koch A, Trautwein C. Liver — guardian, modifier and target of sepsis. Nat Rev Gastroenterol Hepatol. 2017;14(1):55–66. doi: 10.1038/nrgastro.2016.168. 27924081

[5] Longo VD, Panda S. Fasting, circadian rhythms, and time-restricted feeding in healthy lifespan. Cell Metab. 2016;23(6):1048–1059. doi: 10.1016/j.cmet.2016.06.001. 27304506 PMC5388543

[6] Teong XT, Liu K, Vincent AD, Bensalem J, Liu B, Hattersley KJ, Zhao L, Feinle-Bisset C, Sargeant TJ, Wittert GA, et al. Intermittent fasting plus early time-restricted eating versus calorie restriction and standard care in adults at risk of type 2 diabetes: a randomized controlled trial. Nat Med. 2023;29(4):963–972. doi: 10.1038/s41591-023-02287-7. 37024596

[7] Sutton EF, Beyl R, Early KS, Cefalu WT, Ravussin E, Peterson CM. Early time-restricted feeding improves insulin sensitivity, blood pressure, and oxidative stress even without weight loss in men with prediabetes. Cell Metab. 2018;27(6):1212–21.e3. doi: 10.1016/j.cmet.2018.04.010. 29754952 PMC5990470

[8] Chaix A, Lin T, Le HD, Chang MW, Panda S. Time-restricted feeding prevents obesity and metabolic syndrome in mice lacking a circadian clock. Cell Metab. 2019;29(2):303–19.e4. doi: 10.1016/j.cmet.2018.08.004. 30174302 PMC7751278

[9] Sommer F, Anderson JM, Bharti R, Raes J, Rosenstiel P. The resilience of the intestinal microbiota influences health and disease. Nat Rev Microbiol. 2017;15(10):630–638. doi: 10.1038/nrmicro.2017.58. 28626231

[10] Adelman MW, Woodworth MH, Langelier C, Busch LM, Kempker JA, Kraft CS, Martin GS. The gut microbiome’s role in the development, maintenance, and outcomes of sepsis. Crit Care. 2020;24:278. 32487252 10.1186/s13054-020-02989-1 PMC7266132

[11] Xie S, Li J, Lyu F, Xiong Q, Gu P, Chen Y, Chen M, Bao J, Zhang X, Wei R, et al. Novel tripeptide RKH derived from Akkermansia muciniphila protects against lethal sepsis. Gut. 2024;73(1):78–91. doi: 10.1136/gutjnl-2023-329996. 37553229

[12] Gu P, Liu R, Yang Q, Xie L, Wei R, Li J, Mei F, Chen T, Zeng Z, He Y, Zhou H. A metabolite from commensal Candida albicans enhances the bactericidal activity of macrophages and protects against sepsis. Cell Mol Immunol. 2023;20:1156–1170. 37553429 10.1038/s41423-023-01070-5 PMC10541433

[13] Shi H, Zhang B, Abo-Hamzy T, Nelson JW, Ambati CSR, Petrosino JF, Bryan RM, Durgan DJ. Restructuring the gut microbiota by intermittent fasting lowers blood pressure. Circ Res. 2021;128(9):1240–1254. doi: 10.1161/CIRCRESAHA.120.318155. 33596669 PMC8085162

[14] Liu Z, Dai X, Zhang H, Shi R, Hui Y, Jin X, Zhang W, Wang L, Wang Q, Wang D, et al. Gut microbiota mediates intermittent-fasting alleviation of diabetes-induced cognitive impairment. Nat Commun. 2020;11(1):855. doi: 10.1038/s41467-020-14676-4. 32071312 PMC7029019

[15] Pan F, Zhang L, Li M, Hu Y, Zeng B, Yuan H, Zhao L, Zhang C. Predominant gut lactobacillus murinus strain mediates anti-inflammaging effects in calorie-restricted mice. Microbiome. 2018;6(1):54. doi: 10.1186/s40168-018-0440-5. 29562943 PMC5863386

[16] Wilck N, Matus MG, Kearney SM, Olesen SW, Forslund K, Bartolomaeus H, Haase S, Mähler A, Balogh A, Markó L, et al. Salt-responsive gut commensal modulates TH17 axis and disease. Nature. 2017;551(7682):585–589. doi: 10.1038/nature24628. 29143823 PMC6070150

[17] Tang C, Kamiya T, Liu Y, Kadoki M, Kakuta S, Oshima K, Hattori M, Takeshita K, Kanai T, Saijo S, et al. Inhibition of dectin-1 signaling ameliorates colitis by inducing lactobacillus-mediated regulatory T cell expansion in the intestine. Cell Host & Microbe. 2015;18(2):183–197. doi: 10.1016/j.chom.2015.07.003. 26269954

[18] Okada T, Fukuda S, Hase K, Nishiumi S, Izumi Y, Yoshida M, Hagiwara T, Kawashima R, Yamazaki M, Oshio T, Otsubo T. Microbiota-derived lactate accelerates colon epithelial cell turnover in starvation-refed mice. Nat Commun. 2013;4:1654. 23552069 10.1038/ncomms2668

[19] Hu J, Deng F, Zhao B, Lin Z, Sun Q, Yang X, Wu M, Qiu S, Chen Y, Yan Z, et al. Lactobacillus murinus alleviate intestinal ischemia/reperfusion injury through promoting the release of interleukin-10 from M2 macrophages via Toll-like receptor 2 signaling. Microbiome. 2022;10(1):38. doi: 10.1186/s40168-022-01227-w. 35241180 PMC8896269

[20] Singer JR, Blosser EG, Zindl CL, Silberger DJ, Conlan S, Laufer VA, DiToro D, Deming C, Kumar R, Morrow CD, Segre JA. Preventing dysbiosis of the neonatal mouse intestinal microbiome protects against late-onset sepsis. Nat Med. 2019;25:1772–1782. 31700190 10.1038/s41591-019-0640-y PMC7250008

[21] Chakraborty S, Galla S, Cheng X, Yeo JY, Mell B, Singh V, Yeoh B, Saha P, Mathew AV, Vijay-Kumar M, et al. Salt-responsive metabolite, β-hydroxybutyrate, attenuates hypertension. Cell Rep. 2018;25(3):677–89.e4. doi: 10.1016/j.celrep.2018.09.058. 30332647 PMC6542293

[22] Leclercq S, Le Roy T, Furgiuele S, Coste V, Bindels LB, Leyrolle Q, Neyrinck AM, Quoilin C, Amadieu C, Petit G, Dricot L. Gut microbiota-induced changes in β-Hydroxybutyrate metabolism are linked to altered sociability and depression in alcohol use disorder. Cell Rep. 2020;33:108238. 33053357 10.1016/j.celrep.2020.108238

[23] Tajima T, Yoshifuji A, Matsui A, Itoh T, Uchiyama K, Kanda T, Tokuyama H, Wakino S, Itoh H. β-hydroxybutyrate attenuates renal ischemia-reperfusion injury through its anti-pyroptotic effects. Kidney Int. 2019;95(5):1120–1137. doi: 10.1016/j.kint.2018.11.034. 30826015

[24] Jiang S, Zhu W, Li C, Zhang X, Lu T, Ding Z, Cao K, Liu L. α-Lipoic acid attenuates lps-induced cardiac dysfunction through a PI3K/Akt-dependent mechanism. Int Immunopharmacol. 2013;16:100–107. 23562296 10.1016/j.intimp.2013.03.024

[25] Zhao Y, Feng X, Li B, Sha J, Wang C, Yang T, Cui H, Fan H. Dexmedetomidine protects against lipopolysaccharide-induced acute kidney injury by enhancing autophagy through inhibition of the PI3K/AKT/mTOR Pathway. Front Pharmacol. 2020;11:128. 32158395 10.3389/fphar.2020.00128 PMC7052304

[26] Chen L, Liu P, Feng X, Ma C. Salidroside suppressing lps-induced myocardial injury by inhibiting ros-mediated PI3K/Akt/mTOR pathway in vitro and in vivo. J Cellular Mol Medi. 2017;21(12):3178–3189. doi: 10.1111/jcmm.12871. PMC5706507 28905500

[27] Yang Y, Tian T, Li S, Li N, Luo H, Jiang Y. LncRNA 220: a novel long non-coding RNA regulates autophagy and apoptosis in Kupffer Cells via the miR-5101/PI3K/AKT/mTOR axis in LPS-Induced endotoxemic liver injury in mice. IJMS. 2023;24(13):24. doi: 10.3390/ijms241311210. PMC10342868 37446388

[28] Bi CF, Liu J, Hao SW, Xu ZX, Ma X, Kang XF, Yang LS, Zhang JF. Xuebijing injection protects against sepsis-induced myocardial injury by regulating apoptosis and autophagy via mediation of PI3K/AKT/mTOR signaling pathway in rats. Aging (Albany NY). 2023;15:4374–4390. 37219401 10.18632/aging.204740 PMC10258031

[29] Huffman TA, Mothe-Satney I, Lawrence JC Jr. Insulin-stimulated phosphorylation of lipin mediated by the mammalian target of rapamycin. Proc Natl Acad Sci USA. 2002;99(2):1047–1052. doi: 10.1073/pnas.022634399. 11792863 PMC117427

[30] Dai W, Zheng P, Luo D, Xie Q, Liu F, Shao Q, Zhao N, Qian K. LPIN1 is a regulatory factor associated with immune response and inflammation in sepsis. Front Immunol. 2022;13:820164. 35222395 10.3389/fimmu.2022.820164 PMC8865371

[31] Zhang A, Yang J, Ma C, Li F, Luo H. Development and validation of a robust ferroptosis-related prognostic signature in lung adenocarcinoma. Front Cell Dev Biol. 2021;9:616271. doi: 10.3389/fcell.2021.616271. 34249899 PMC8264775

[32] Zhou Z, Ye TJ, Bonavita G, Daniels M, Kainrad N, Jogasuria A, You M. Adipose-specific lipin-1 overexpression renders hepatic ferroptosis and exacerbates alcoholic steatohepatitis in mice. Hepatol Commun. 2019;3(5):656–669. doi: 10.1002/hep4.1333. 31061954 PMC6492478

[33] Wang J, Zhu Q, Li R, Zhang J, Ye X, Li X. YAP1 protects against septic liver injury via ferroptosis resistance. Cell Biosci. 2022;12:163. 36182901 10.1186/s13578-022-00902-7 PMC9526934

[34] Zheng Y, Sun W, Shan C, Li B, Liu J, Xing H, Xu Q, Cui B, Zhu W, Chen J, Liu L. β-hydroxybutyrate inhibits ferroptosis-mediated pancreatic damage in acute liver failure through the increase of H3K9bhb. Cell Rep. 2022;41:111847. 36543135 10.1016/j.celrep.2022.111847

[35] Cui X, Yun X, Sun M, Li R, Lyu X, Lao Y, Qin X, Yu W. HMGCL-induced β-hydroxybutyrate production attenuates hepatocellular carcinoma via DPP4-mediated ferroptosis susceptibility. Hepatol Int. 2023;17(2):377–392. doi: 10.1007/s12072-022-10459-9. 36508088 PMC10119270

[36] Caporaso JG, Kuczynski J, Stombaugh J, et al. QIIME allows analysis of high-throughput community sequencing data. Methods. 2010;7(5):335–6. 10.1038/nmeth.f.303 PMC3156573 20383131

[37] Datta G, Fuller BJ, Davidson BR. Molecular mechanisms of liver ischemia reperfusion injury: insights from transgenic knockout models. World J Gastroenterol. 2013;19:1683–1698. 23555157 10.3748/wjg.v19.i11.1683 PMC3607745

[38] Gu J, Sun P, Zhao H, Watts HR, Sanders RD, Terrando N, Xia P, Maze M, Ma D. Dexmedetomidine provides renoprotection against ischemia-reperfusion injury in mice. Crit Care. 2011;15(3):R153. doi: 10.1186/cc10283. 21702944 PMC3219027

[39] Koksel O, Ozdulger A, Ercil M, Tamer L, Ercan B, Atik U, Cinel L, Cinel I, Kanik A. Effects of N -Acetylcysteine on oxidant-antioxidant balance in oleic acid–induced lung injury. Exp Lung Res. 2004;30(6):431–446. doi: 10.1080/01902140490476319. 15524403

[40] Kimura I, Inoue D, Maeda T, Hara T, Ichimura A, Miyauchi S, Kobayashi M, Hirasawa A, Tsujimoto G. Short-chain fatty acids and ketones directly regulate sympathetic nervous system via G protein-coupled receptor 41 (GPR41). Proc Natl Acad Sci USA. 2011;108:8030–8035. 21518883 10.1073/pnas.1016088108 PMC3093469

[41] Zhang SJ, Li ZH, Zhang YD, Chen J, Li Y, Wu FQ, Wang W, Cui ZJ, Chen GQ. Ketone body 3-hydroxybutyrate ameliorates atherosclerosis via receptor Gpr109a-mediated calcium influx. Adv Sci (Weinh). 2021;8:2003410. 33977048 10.1002/advs.202003410 PMC8097358

[42] Huang M, Yu Y, Tang X, Dong R, Li X, Li F, Jin Y, Gong S, Wang X, Zeng Z, et al. 3-hydroxybutyrate ameliorates sepsis-associated acute lung injury by promoting autophagy through the activation of GPR109α in macrophages. Biochemical Pharmacol. 2023;213:115632. doi: 10.1016/j.bcp.2023.115632. 37263300

[43] Asif S, Kim RY, Fatica T, Sim J, Zhao X, Oh Y, Denoncourt A, Cheung AC, Downey M, Mulvihill EE., Kim KH. Hmgcs2-mediated ketogenesis modulates high-fat diet-induced hepatosteatosis. Mol Metab. 2022;61:101494. 35421611 10.1016/j.molmet.2022.101494 PMC9039870

[44] Wang YH, Suk FM, Liao YJ. Loss of HMGCS2 enhances lipogenesis and attenuates the protective effect of the ketogenic diet in liver cancer. Cancers (Basel). 2020;12(7):12. doi: 10.3390/cancers12071797. PMC7408319 32635582

[45] Zou XZ, Hao JF, Hou MX. Hmgcs2 regulates M2 polarization of macrophages to repair myocardial injury induced by sepsis. Aging (Albany NY). 2023;15:7794–7810. doi: 10.18632/aging.204944. 37561521 PMC10457052

[46] Wang Q, Zhou Y, Rychahou P, Fan TW, Lane AN, Weiss HL, Evers BM. Ketogenesis contributes to intestinal cell differentiation. Cell Death Differ. 2017;24(3):458–468. doi: 10.1038/cdd.2016.142. 27935584 PMC5344206

[47] Chen HC, Liu YW, Chang KC, Wu YW, Chen YM, Chao YK, You MY, Lundy DJ, Lin CJ, Hsieh ML, Cheng YC. Gut butyrate-producers confer post-infarction cardiac protection. Nat Commun. 2023;14:7249. 37945565 10.1038/s41467-023-43167-5 PMC10636175

[48] Tripathi A, Debelius J, Brenner DA, Karin M, Loomba R, Schnabl B, Knight R. Publisher correction: the gut–liver axis and the intersection with the microbiome. Nat Rev Gastroenterol Hepatol. 2018;15(12):785. doi: 10.1038/s41575-018-0031-8. 29785003 PMC7133393

[49] Li XY, Yu ZL, Zhao YC, Wang DD, Xue CH, Zhang TT, Wang YM. Gut microbiota metabolite TMA May mediate the effects of TMAO on glucose and lipid metabolism in C57BL/6J mice. Mol Nutr Food Res. 2024;68:e2300443. 38456781 10.1002/mnfr.202300443

[50] Kim MJ, Kim YS, Kim SR, Lee DW, Lee SB, Kim IY. β-hydroxybutyrate ameliorates sepsis-induced acute kidney injury. Mol Biol Rep. 2023;50:8915–8923. 37704932 10.1007/s11033-023-08713-w

[51] Wang X, Song Y, Chen J, Zhang S, Le Y, Xie Z, Ouyang W, Tong J. Subcutaneous administration of β-hydroxybutyrate improves learning and memory of sepsis surviving mice. Neurotherapeutics. 2020;17(2):616–626. doi: 10.1007/s13311-019-00806-4. 31853744 PMC7283433

[52] Faraco G, Hochrainer K, Segarra SG, Schaeffer S, Santisteban MM, Menon A, Jiang H, Holtzman DM, Anrather J, Iadecola C. Dietary salt promotes cognitive impairment through tau phosphorylation. Nature. 2019;574:686–690. 31645758 10.1038/s41586-019-1688-z PMC7380655

[53] Zhang SJ, Li ZH, Zhang YD, et al. Ketone body 3-hydroxybutyrate ameliorates atherosclerosis via receptor gpr109a-mediated calcium influx. Adv Sci (Weinh). 2021;8(9):2003410. 33977048 10.1002/advs.202003410 PMC8097358

[54] Weckx R, Goossens C, Derde S, Pauwels L, Vander Perre S, Van den Bergh G, Langouche L. Identification of the toxic threshold of 3-hydroxybutyrate-sodium supplementation in septic mice. BMC Pharmacol Toxicol. 2021;22:50. 34544493 10.1186/s40360-021-00517-7 PMC8454128

[55] Kashiwagi S, Kanda N, Yoshida M, Wakimoto Y, Ohbe H, Nakamura K. Effects of early enteral nutrition on persistent inflammation, immunosuppression, and catabolism syndrome in critically ill patients: a claims database study using a propensity score analysis. Clin Nutr. 2024;43:1872–1879. 38968719 10.1016/j.clnu.2024.06.033

[56] Geng H, Zhang H, Cheng L, Dong S. Sivelestat ameliorates sepsis-induced myocardial dysfunction by activating the PI3K/AKT/mTOR signaling pathway. Int Immunopharmacol. 2024;128:111466. doi: 10.1016/j.intimp.2023.111466. 38176345

[57] Hu Y, Han J, Ding S, Liu S, Wang H. Identification of ferroptosis-associated biomarkers for the potential diagnosis and treatment of postmenopausal osteoporosis. Front Endocrinol. 2022;13:986384. doi: 10.3389/fendo.2022.986384. PMC9464919 36105394

[58] Yan Q, Tang S, Zhou C, Han X, Tan Z. Effects of free fatty acids with different chain lengths and degrees of saturability on the milk Fat synthesis in primary cultured bovine mammary epithelial cells. J Agric Food Chem. 2019;67:8485–8492. 31304752 10.1021/acs.jafc.9b02905

[59] Lin S, Wang L, Jia Y, Sun Y, Qiao P, Quan Y, Liu J, Hu H, Yang B, Zhou H. Lipin-1 deficiency deteriorates defect of fatty acid β-oxidation and lipid-related kidney damage in diabetic kidney disease. Transl Res. 2024;266:1–15. 37433392 10.1016/j.trsl.2023.07.004

[60] Zheng YN, Lou SY, Lu J, Zheng FL, Tang YM, Zhang EJ, Cui SL, Zhao HJ. Selective PI3Kδ inhibitor TYM-3-98 suppresses AKT/mTOR/SREBP1-mediated lipogenesis and promotes ferroptosis in kras-mutant colorectal cancer. Cell Death Dis. 2024;15:474. 38956060 10.1038/s41419-024-06848-7 PMC11220027

[61] Huang X, Xia K, Wei Z, Liu W, Wei Z, Guo W. SLC38A5 suppresses ferroptosis through glutamine-mediated activation of the PI3K/AKT/mTOR signaling in osteosarcoma. J Transl Med. 2024;22(1):1004. doi: 10.1186/s12967-024-05803-6. 39511570 PMC11542360

[62] Xie L, Zhou C, Wu Y, Fu X, Zhang G, Han X, Xie S, Chen G, Xu H, Deng B, Liu B. Wenqingyin suppresses ferroptosis in the pathogenesis of sepsis-induced liver injury by activating the Nrf2-mediated signaling pathway. Phytomedicine. 2023;114:154748. 36933519 10.1016/j.phymed.2023.154748

[63] Wei S, Bi J, Yang L, Zhang J, Wan Y, Chen X, Wang Y, Wu Z, Lv Y, Wu R. Serum irisin levels are decreased in patients with sepsis, and exogenous irisin suppresses ferroptosis in the liver of septic mice. Clin Transl Med. 2020;10:e173. 32997405 10.1002/ctm2.173 PMC7522760

[64] Xiao Y, Yu Y, Hu L, Yang Y, Yuan Y, Zhang W, Luo J, Yu L. Matrine alleviates sepsis-induced myocardial injury by inhibiting ferroptosis and apoptosis. Inflammation. 2023;46:1684–1696. 37219694 10.1007/s10753-023-01833-2

[65] Kuwata H, Nakatani E, Tomitsuka Y, Ochiai T, Sasaki Y, Yoda E, Hara S. Deficiency of long-chain acyl-CoA synthetase 4 leads to lipopolysaccharide-induced mortality in a mouse model of septic shock. FASEB J. 2023;37:e23330. 37983658 10.1096/fj.202301314R

[66] Li B, Wang Z, Yuan J, Liang D, Cheng Y, Wang Z. Knockdown of SHP2 attenuated lps-induced ferroptosis via downregulating ACSL4 expression in acute lung injury. Allergol Immunopathol (Madr). 2023;51:143–152. 37169572 10.15586/aei.v51i3.856

[67] Gao S, Sun C, Kong J. Vitamin D attenuates ulcerative colitis by inhibiting ACSL4-mediated ferroptosis. Nutrients. 2023;15(22):15. doi: 10.3390/nu15224845. PMC10675831 38004239

