# Supplementary material for: Time-restricted feeding protects against septic liver injury by reshaping gut microbiota and metabolite 3-hydroxybutyrate
Source: Gut Microbes. 2025 Apr 13;17(1):2486515. doi: 10.1080/19490976.2025.2486515 (PMC12005432; doi:10.1080/19490976.2025.2486515)
Supplement: Supplemental Material [file KGMI_A_2486515_SM7129.pdf]

Supplemental information

Figure S1

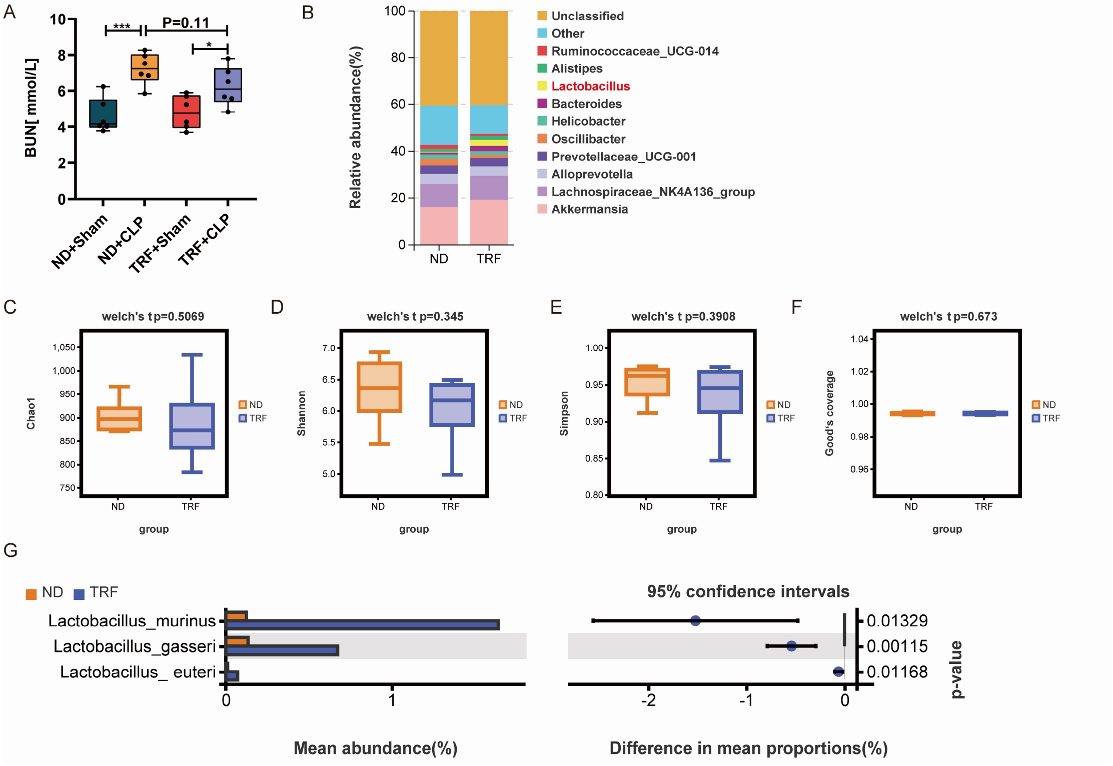

Fig.S1. Effect of TRF on BUN levels, intestinal flora α diversity and flora composition in mice. **(A)** BUN content in serum of mice, n = 6-8. **(B)** Species distribution at the genus level, n = 6. **(C-F)** Microbial α diversity of Chao1, Shannon, Simpson and goods_coverage index, n = 6. **(G)** Statistical test analysis of indicator species at the species level, n = 6. The results are expressed as the median and quartile. * $p < 0.05$, ** $p < 0.01$, *** $p < 0.001$ by wetch's t test.

Figure S2

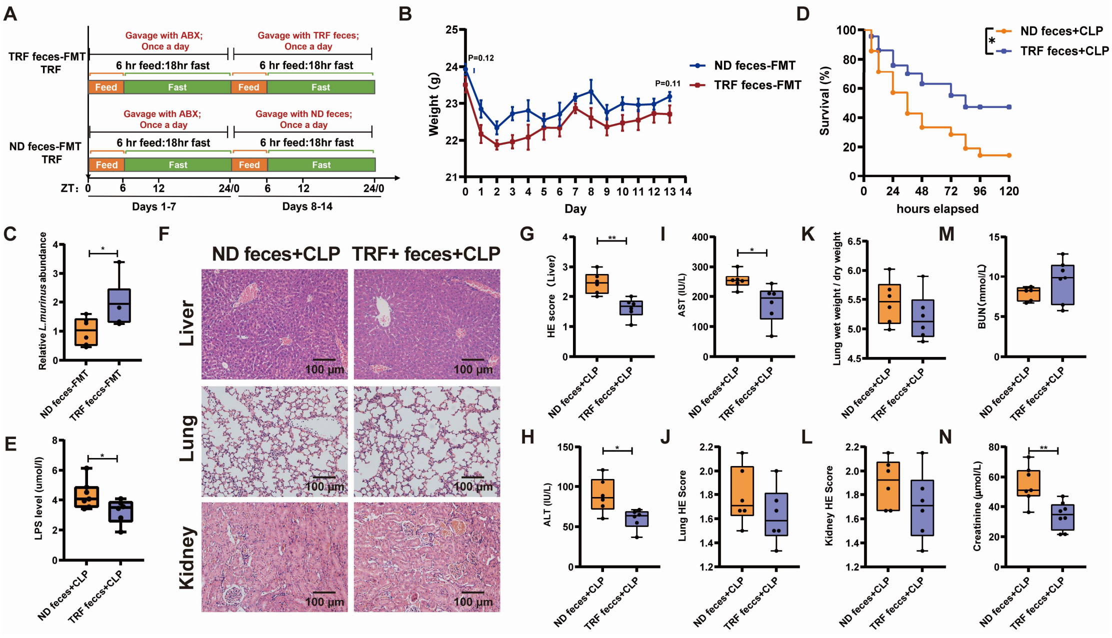

Fig.S2. FMT experiment demonstrated that TRF alleviates sepsis injury via intestinal flora. (A) Preoperative fecal bacteria from ND and TRF mice were transplanted into pseudo-GF mice. **(B)** Weight changes of mice in the ND feces-FMT group and TRF feces-FMT group within 14 days, n = 6-8. **(C)** Relative abundance of *L. murinus* in the feces of mice in the ND feces-FMT and TRF feces-FMT groups was detected by qPCR, n = 6-8. **(D)** 5-day survival of mice (n = 20). **(E)** Serum LPS levels, n = 6-8. **(F)** HE staining of liver, lung and kidney tissues of mice, scale: 100 μm, n = 6. **(G)** Liver histopathological damage scores, n = 6. **(H, I)** Serum ALT and AST levels, n = 6-8. **(J)** Lung histopathological damage scores, n = 6. **(K)** Mouse lung wet/dry weight ratio, n = 6. **(L)** Kidney histopathological damage scores, n = 6. **(M)** Serum BUN levels, n = 6-8. **(N)** Serum Creatinine levels, n = 6-8. The results are expressed as the mean ± SEM (B) and the median and quartile. * $p < 0.05$, ** $p < 0.01$, *** $p < 0.001$ by Log-Rank test (D) and wetch’s t test.

Figure S3

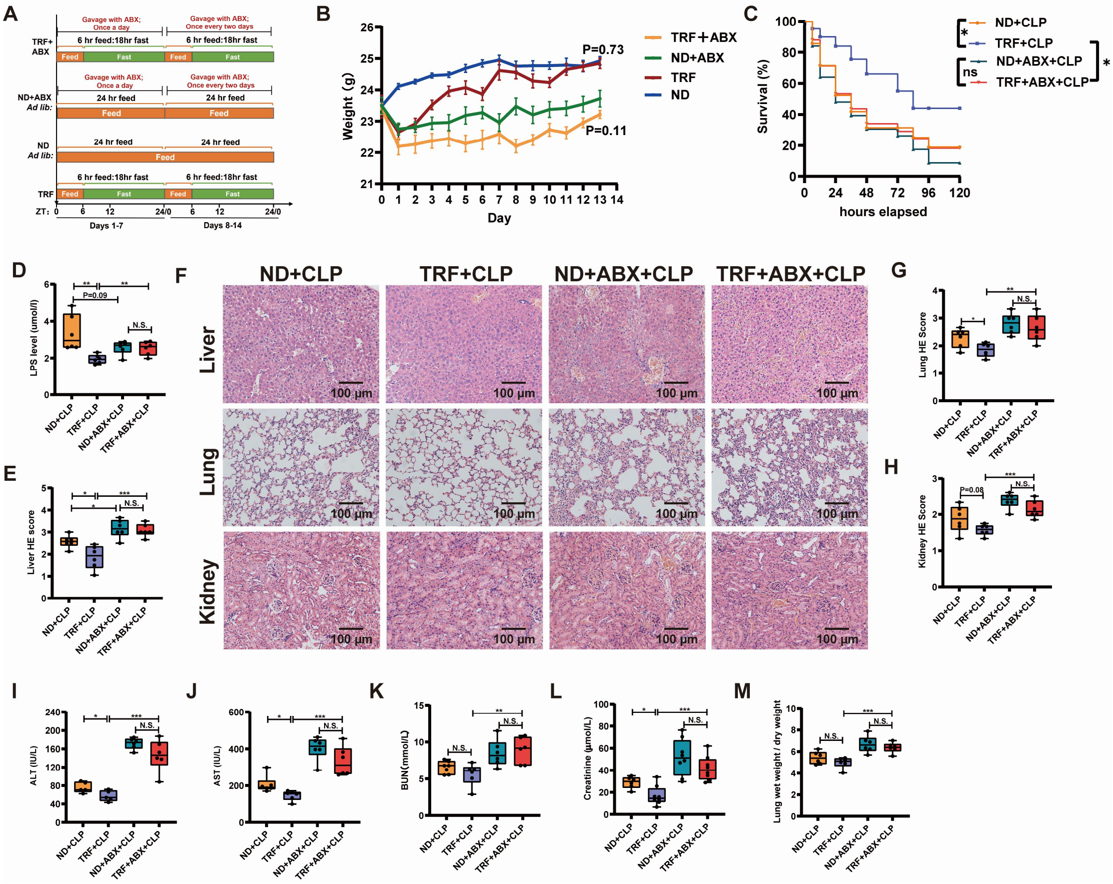

Fig.S3. ABX experiment demonstrated that TRF dependent on intestinal flora attenuates septic liver injury. **(A)** A graph illustrating of the ABX and TRF pattern. **(B)** Weight changes of mice in the TRF+ABX group, ND+ABX group, TRF group and ND group within 14 days, n = 6-8. **(C)** 5-day survival of mice (n = 20). **(D)** LPS content in serum of mice, n = 6-8. **(E)** Relative quantitative map of liver histopathological damage score in mice, n = 6. **(F)** HE staining of liver, lung and kidney tissues of mice, scale: 100 μm, n = 6. **(G, H)** Relative quantitative map of lung and kidney histopathological damage score in mice, n = 6. **(I-L)** ALT, AST, BUN and Creatinine levels in serum of mice, n = 6-8. **(M)** Mouse lung wet/dry weight ratio, n = 6. The results are expressed as the mean ± SEM (B) and the median and quartile. * $p < 0.05$, ** $p < 0.01$, *** $p < 0.001$ by Log-Rank test (C) and two-way ANOVA (Tukey's test).

Figure S4

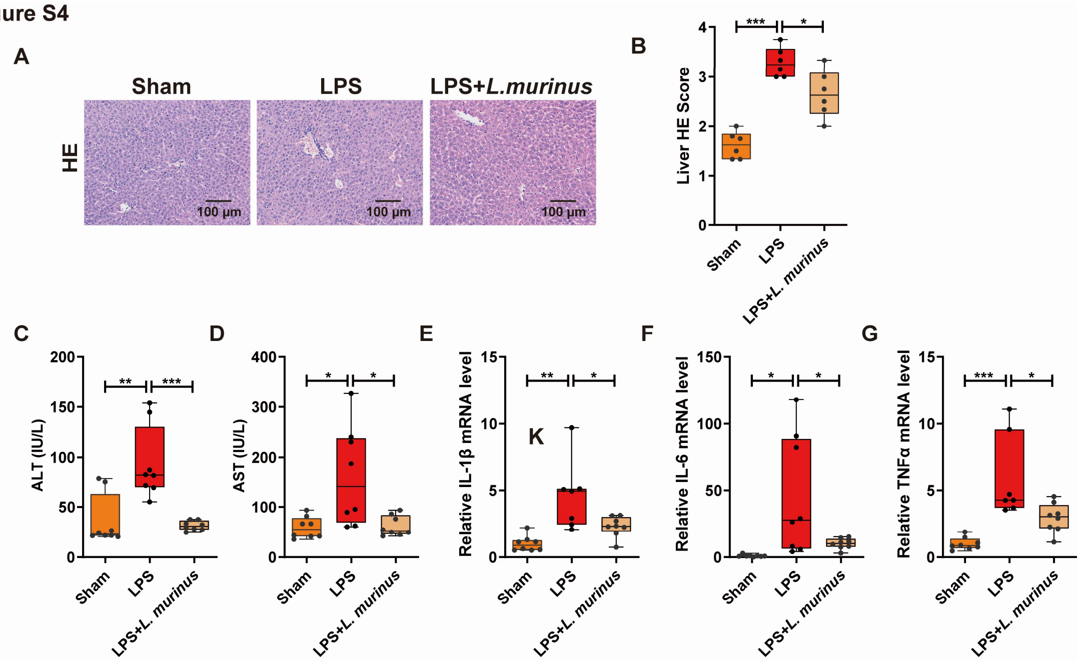

Fig. S4. *L. murinus* can effectively mitigate LPS-induced septic liver injury. (A, B) HE staining and quantification analysis in liver, scale: 100 μm, n = 6. **(C, D)** Serum ALT and AST levels in mice, n = 6-8. **(E-G)** Relative mRNA expression of *Il-1β*, *Il-6*, and *Tnf-α* in liver tissues of mice, n = 6-8. The results are expressed as the median and quartile. * $p < 0.05$, ** $p < 0.01$, *** $p < 0.001$ by one-way ANOVA (Tukey's test).

Figure S5

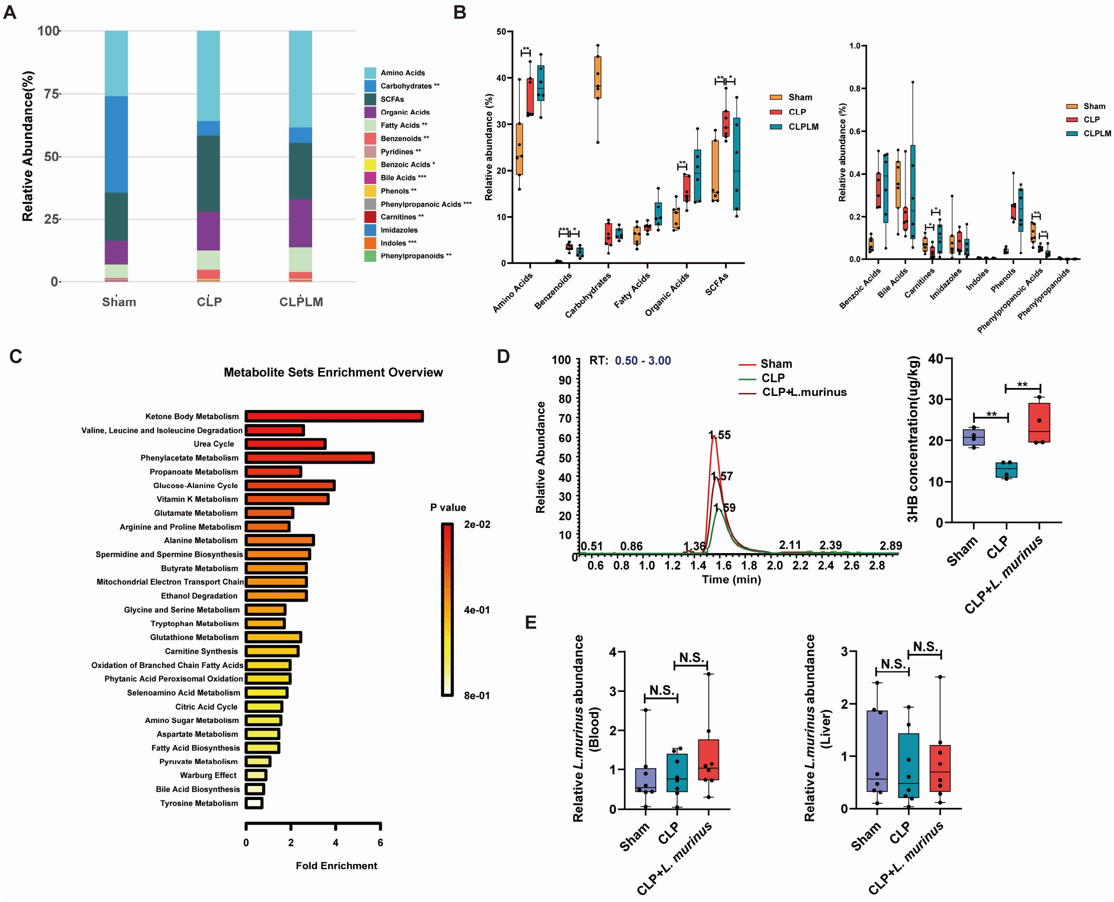

Fig.S5. Q200-targeted metabolomics analysis. (A, B) Relative abundances of various metabolites in samples from Sham group, CLP group and CLP+*L. murinus* (CLPLM) group, n = 6-8. **(C)** Enrichment analysis of differential metabolite pathways, n = 6-8. **(D)** 3-HB content in postoperative cecum contents from Sham, CLP, and CLP+*L. murinus* groups, n = 4. **(E)** Relative abundance of *L. murinus* in blood and liver tissues detected by qPCR, n = 6-8.

Figure S6

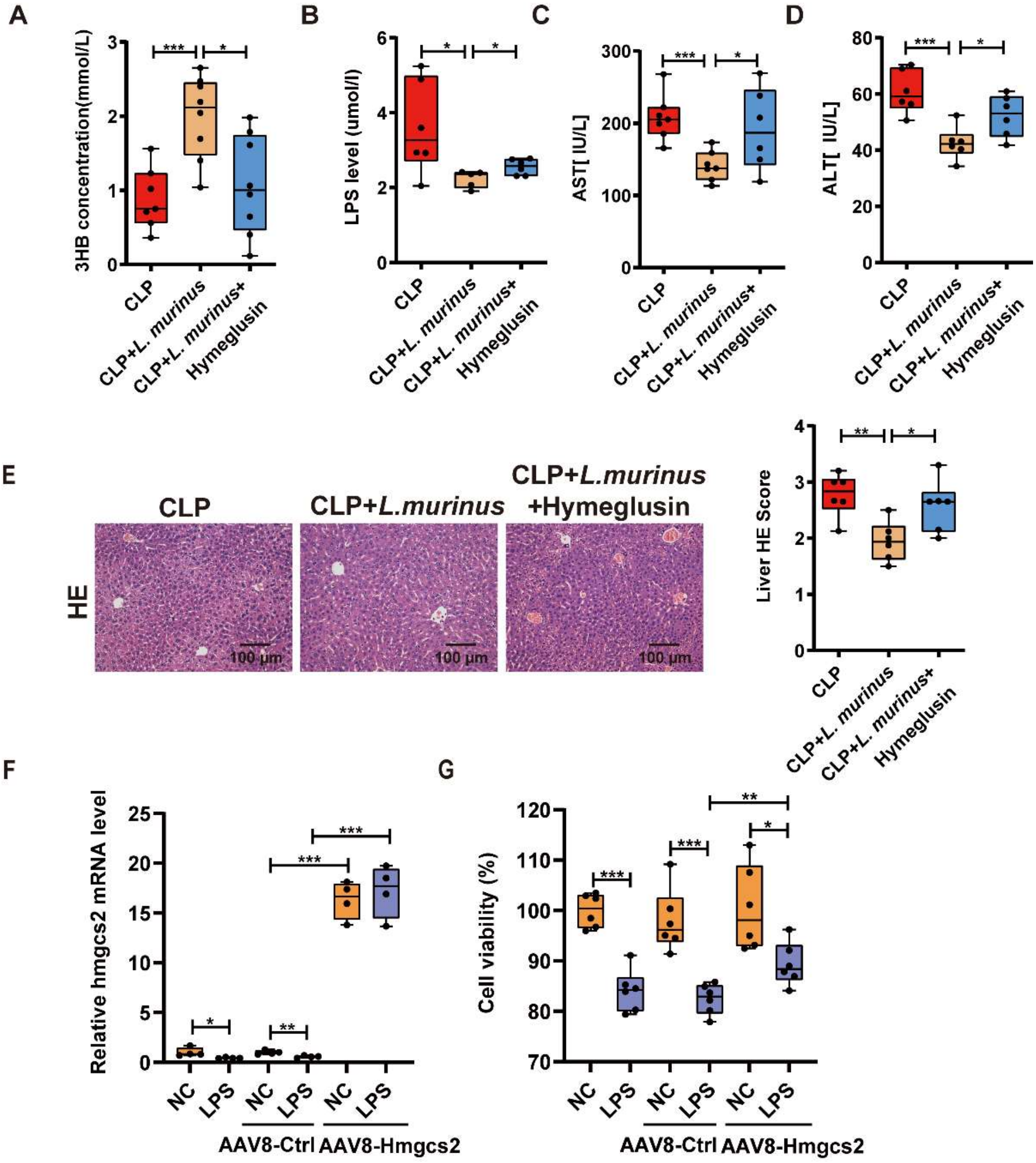

Fig.S6. Hmgcs2 enzyme inhibitor hymeglusin abolishes the protection of *L. murinus* against septic liver injury. (A) 3-HB content in serum of mice in the CLP group, CLP+*L. murinus* group and CLP+*L. murinus*+ Hymeglusin group, n = 6-8. **(B)** LPS content in serum of mice, n = 6-8. **(C, D)** AST and ALT content in serum of mice, n = 6-8. **(E)** HE staining in the liver and the quantification analysis, scale: 100 μm, n = 6. **(F)** AAV-Hmgcs2 transfection increased Hmgcs2 mRNA expression in AML12 cells, n = 5. **(G)** AML12 cell viability was detected by CCk8, n = 6. The results are expressed

as the median and quartile. * $p < 0.05$, ** $p < 0.01$, *** $p < 0.001$ by one-way ANOVA (Tukey's test). AAV: Adeno-Associated Virus; 3-HB: 3- hydroxybutyric acid; *L. murinus*: *Lactobacillus murinus*; ALT: Alanine Aminotransferase; AST: Aspartate aminotransferase.

Figure S7

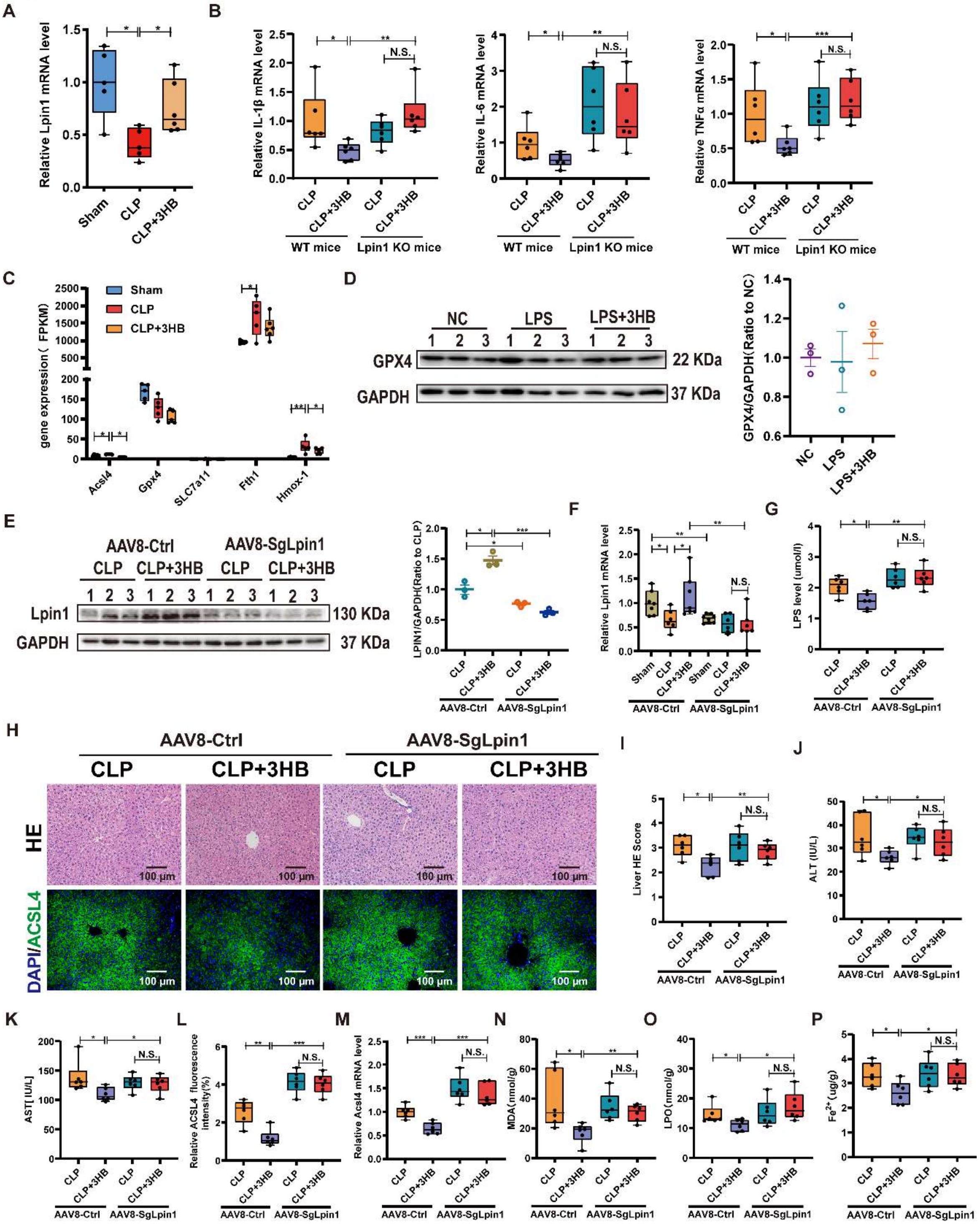

Fig.S7. 3-HB inhibits ferroptosis in mice by activating the PI3K/AKT/mTOR/LPIN1 pathway. (A) Relative Lpin1 mRNA expression, n = 5-6. **(B)** Relative mRNA expression of *I1-1β*, *Il-6*, and *Tnf-α* in liver tissues from WT and Lpin1KO mice, n = 5-6. **(C)** Relative fpkm value expression of *Acsl4*, *Gpx4*, *SLC7a11*,

Fth1 and *Hmox-1* genes, n = 5-6. **(D)** GPX4 protein expression level in the AML12 cells and relative quantitative analysis, n = 3. **(E)** The protein expression levels of liver tissue LPIN1 in the AAV-Ctrl and AAV-SgLpin1 groups (n = 3). **(F)** Relative Lpin1 mRNA expression level, n = 6-8. **(G)** LPS content in serum of mice, n = 6-8. **(H)** HE staining and ACSL4 immunofluorescence of liver tissue, scale: 100 μm, n = 6. **(I)** Relative quantitative map of liver histopathological damage score in mice, n = 6. **(J, K)** ALT and AST content in serum of mice, n = 6-8. **(L)** Relative quantitative analysis of ACSL4 immunofluorescence intensity, n = 6. **(M)** Relative ACSL4 mRNA expression level, n = 6-8. (**N-P**) MDA, LPO and Fe^{2+} content in liver tissue, n = 6. The results are expressed as the median and quartile and mean ± SEM(D, E). * $p < 0.05$， ** $p < 0.01$， *** $p < 0.001$ by one-way ANOVA (Tukey's test)(A, C,D) and two-way ANOVA (Tukey's test)(B, E-P). AAV: Adeno-Associated Virus; 3-HB: 3-hydroxybutyric acid; MDA: Malondialdehyde; LPO: Lipid Peroxidation.

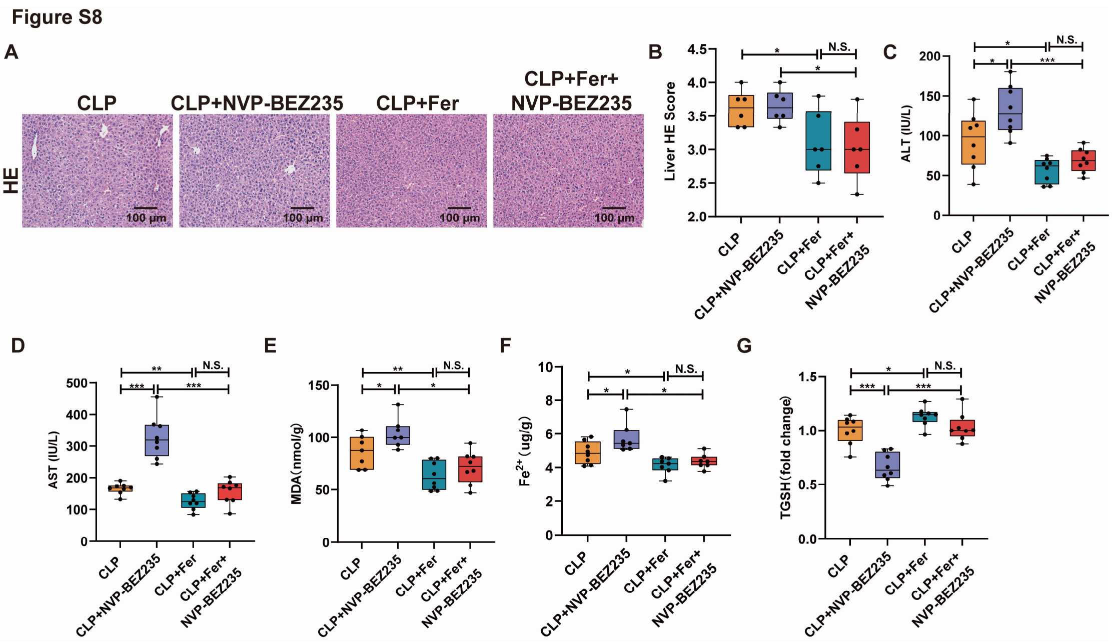

Fig.S8. NVP-BEZ235 protects against septic liver injury by inhibiting ferroptosis. **(A, B)** HE staining and quantification analysis in liver, scale: 100 μm, n = 6. **(C, D)** Serum ALT and AST levels in mice, n = 6-8. **(E-G)** MDA, $Fe^{2+}$and TGSH content in liver tissue, n = 6. The results are expressed as the median and quartile. * $p < 0.05$, ** $p < 0.01$, *** $p < 0.001$ by two-way ANOVA (Tukey's test).

Figure S9

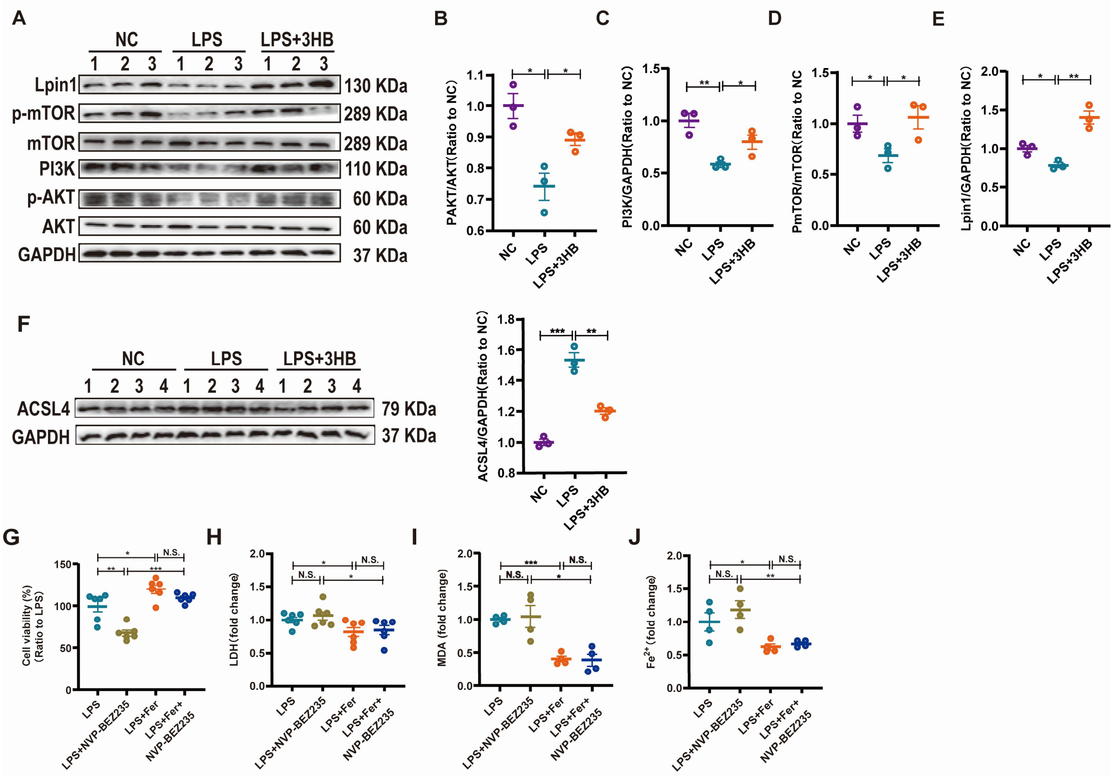

Fig.S9. 3-HB activates the PI3K/AKT/mTOR/LPIN1 Pathway to suppress ferroptosis in AML12 Cells. (A-F) The protein expression levels of AKT, p-AKT, PI3K, mTOR, p-mTOR, LPIN1 and ACSL4 in the AML12 cells were detected by Western blot (n = 3). **(G)** AML12 cell viability by CCk8, n = 6. **(H)** LDH content in AML12 cell supernatant, n = 6. **(I, J)** MDA and Fe^{2+} content in the AML12 cells, n = 4. The results are expressed as the mean ± SEM. * $p < 0.05$, ** $p < 0.01$, *** $p < 0.001$ by one-way ANOVA (Tukey's test) and two-way ANOVA (Tukey's test).

Table S1 Quantitative RT-PCR primer sequence.

Table S2 Quantitative RT-PCR primer sequence

Gene	Forward primer (5'–3')	Reverse primer (5'–3')
18S	CGATCCGAGGGCCTCACTA	AGTCCCTGCCCTTTGTACACA
Hmgcs2	TCAGGGGTCTAAAGCTGGAA	TAAGCCTGAGCCGTAGGAGA
Lpin1	CTCCGCTCCCGAGAGAAAG	TCATGTGCAAATCCACGGACT
Gpx4	TGTGCATCCCGCGATGATT	CCCTGTACTTATCCAGGCAGA
Acsl4	CCTGAGGGGCTTGAAATTCA C	GTTGGTCTACTTGGAGGAACG
SLC7a11	GGCACCGTCATCGGATCAG	CTCCACAGGCAGACCAGAAA A
Fth1	CAAGTGCGCCAGAACTACCA	ACAGATAGACGTAGGAGGCA TAC
Hmox-1	AGGTACACATCCAAGCCGA GA	CATCACCAGCTTAAAGCCTTC T
16S	GTGSTGCAYGGYTGTCGTCA	ACGTCRTCCMCACCTTCCTC

Table S2. The characteristics of patients with sepsis. Measurement data are expressed as (x±s) and t-test was performed; count data are expressed as (%) and χ2 test was performed for intergroup comparison.

Table S1 The characteristics of patients with sepsis

Factors	SLI(n=17)	SNLI(n=40)	t/χ2	*p* value
Male	11(64.71%)	25(62.50%)	0.025	0.87
Female	6(35.29%)	15(37.50%)	0.025	0.87
Age(years)	63.76±11.66	68.83±11.65	1.5	0.14
SOFA	6.18±3.07	5.65±3.13	0.58	0.56
APACHE II	20.71±5.50	19.23±5.20	0.97	0.34
Lactate(mmol/l)	2.70±3.92	1.44±0.55	2.02	0.049*
PaO2/FiO2	279.30±116.50	262.70±129.90	0.44	0.66
HGB(g/l)	104.50±27.48	99.10±23.09	0.76	0.45
ALT(U/L)	120.80±75.70	35.73±22.31	6.54	<0.0001
AST(U/L)	120.90±73.74	37.95±23.43	6.46	<0.0001
ICU Time(d)	15.76±10.44	10.32±6.35	2.39	0.021*
TBIL(μmol/l)	18.61±16.96	15.25±15.48	0.73	0.47
INR	1.36±0.60	1.37±0.60	0.06	0.95
